# Biodegradable Microcapsules of Poly(Butylene Adipate-*co*-Terephthalate) (PBAT) as Isocyanate Carriers and the Effect of the Process Parameters

**DOI:** 10.3390/polym15030665

**Published:** 2023-01-28

**Authors:** António Aguiar, António Mariquito, Diogo Gonçalves, Isabel Pinho, Ana C. Marques

**Affiliations:** 1 CERENA, DEQ, Instituto Superior Técnico, Universidade de Lisboa, Avenida Rovisco Pais, 1049-001 Lisboa, Portugal; 2 CIPADE—Indústria e Investigação de Produtos Adesivos, SA., Av. Primeiro de Maio 121, 3700-227 São João da Madeira, Portugal

**Keywords:** Poly(butylene adipate-*co*-terephthalate), PBAT, biodegradable microcapsules, isophorone diisocyanate, IPDI, solvent evaporation, morphology

## Abstract

Poly(butylene adipate-*co*-terephthalate) (PBAT), a biodegradable flexible, and tough polymer is herein used, for the first time, to encapsulate and protect isocyanate derivatives. Isocyanates are essential building blocks widely employed in the chemical industry for the production of high-performing materials. Microencapsulation of isocyanates eliminates the risks associated with their direct handling and protects them from moisture. In light of this, and having in mind eco-innovative products and sustainability, we present a straightforward process to encapsulate isophorone diisocyanate (IPDI) using this biodegradable polymer. Spherical and core-shell microcapsules (MCs) were produced by an emulsion system combined with the solvent evaporation method. The MCs present a regular surface, without holes or cracks, with a thin shell and high isocyanate loadings, up to 79 wt%. Additionally, the MCs showed very good isocyanate protection if not dispersed in organic or aqueous solutions. Effects of various process parameters were systematically studied, showing that a higher stirring speed (1000 rpm) and emulsifier amount (2.5 g), as well as a smaller PBAT amount (1.60 g), lead to smaller MCs and narrower size distribution.

## 1. Introduction

The term “isocyanate” is the generic name of the chemical compound, found in 1848, that is characterized by having at least one chemical group with the formula –R–N=C=O [1]. Due to their reactivity and versatility, isocyanate derivatives are capable of a surprising variety of chemical reactions, and consequently, they have been used in a wide range of industrial products [1,2]. The list of new isocyanates has been growing, but the most employed isocyanates are still the methylene diphenyl isocyanate (MDI), toluene diisocyanate (TDI), hexamethylene diisocyanate (HDI), and isophorone diisocyanate (IPDI). Despite the isocyanates’ useful properties and the significant developments in their employment, the sensitivity of isocyanates towards moisture limits their application and lowers their storage life. Furthermore, isocyanates are strong irritants to the mucous membranes of the eyes and are one of the most reported causes of occupational asthma worldwide [3,4,5].

Microencapsulation is a procedure that has gained notoriety over time and affords some measure of protection in handling hazardous materials, such as isocyanates. The shielding effect of microcapsules (MCs) can greatly improve the protection of isocyanate, which is essential for storage and transportation [6]. Currently, microencapsulation is used in numerous industrial sectors, such as the pharmaceutical, food, textile, cosmetics, and adhesive industries [6,7,8,9,10,11,12,13]. Depending on the shell material, the encapsulated compounds are protected from light, humidity, and interactions with other substances. Nevertheless, the encapsulated compounds can be released through several chemical or physical trigger mechanisms [14,15], being delivered when and where needed.

Microencapsulation is a coating process where individual liquid droplets, solid particles, or gas bubbles are surrounded by a continuous material [16]. Commercial microcapsules (MCs) typically have a diameter in the range of 3–800 μm [17]. Despite the different morphologies and shapes that they can present [15], spherical and core-shell MCs are usually intended. 

Although the myriad techniques that have been used for the preparation of microcapsules, the solvent evaporation technique is one of the most relevant methods since it is simple, not expensive, and is consistent with the encapsulation of hydrophobic agents, using an oil-in-water emulsion systems (simple emulsion), or hydrophilic agents, using water-in-oil-in-water emulsions (double emulsion). In the solvent evaporation mechanism, for a simple oil-in-water emulsion, the polymer is dissolved in a water-immiscible volatile organic solvent which then dissolves or suspends the compound to be encapsulated. The resultant solution is then added to the aqueous solution (continuous phase), containing emulsifier agents, under homogenization to form an emulsion. After the formation of a stable emulsion, the organic solvent is evaporated either by increasing the temperature under reduced pressure and/or by continuous stirring, yielding a dispersion of solid particles. Adjustments and variations of this well-established technique resulted in numerous investigations and reviews in the past years [18,19,20]. The choice of surfactant is crucial to prevent droplet coalescence and control the surface of the resulting particles [21]. It is also known that MCs porosity could be controlled by the rate of evaporation, the ratio of the internal aqueous phase, and the viscosity of the polymer’s solution [22]. Moreover, the geometry of homogenizing tip, as well as the volume and shape of the homogenizer vessel influence the emulsion droplet size [23].

In recent years, more biodegradable polymers have become commercially available and started to be used in MCs production, which has promoted the application of solvent evaporation technique [20]. This is among the most ecologically friendly and promising solutions for MCs production. Environment and health safety, degradability, encapsulation efficiency, and biocompatibility are some features that biodegradable shells offer for a wide range of products. Natural or synthetic biodegradable polymers including ethyl cellulose [24,25], chitosan [26,27], alginate [28,29], polyacrylamide [30,31], poly(lactic acid) [32,33], copolymers of lactic and glycolic acids [34,35], or polycaprolactone [36,37], have been investigated as MCs shell materials for agrochemicals, drugs, or fragrances. However, their use for isocyanate encapsulation has been poorly explored.

The first report on isocyanate microencapsulation dates from 2008 and it describes the encapsulation of IPDI via interfacial polymerization reactions for the formation of polyurethane and/or polyurea shell [38]. Since then, interfacial polymerization has been consolidated as the most used method for isocyanate encapsulation. However, this chemical process rarely offers high encapsulation rates [39]. On the other hand, the solvent evaporation technique enables a high isocyanate load, with no significant polyurethane/polyurea (PU/PUa) formation [40].

Poly(butylene adipate-*co*-terephthalate) (PBAT) is a synthetic polymer, 100% biodegradable, with high elongation at break, and flexibility [41]. It is a biodegradable alternative to low-density polyethylene (LDPE) and other industry standard plastics. It has been used in several applications, such as packaging materials (food wraps), plastic bags, hygiene products, biomedical devices, etc. Nonetheless, in what regards to MCs, only one study has been found in the literature that discusses the use of PBAT as an encapsulating agent for essential oils [42]. 

In this work, we developed new PBAT MCs with high IPDI loading. IPDI was chosen due to its monomeric structure that facilitates the loading quantification, and because it is a reactive healing and cross-linking agent extensively used in abrasion and UV-resistant PU coatings [38,43]. Besides the production of a new isocyanate container, we studied the influence of various parameters on the properties (MCs size, size distribution and morphology) of the final MCs, as well as their thermal properties and IPDI loading and protection. Stirring speed and the weights of the emulsifier (poly(vinyl alcohol), PBAT, IPDI, and organic solvent were the investigated parameters.

## 2. Materials and Methods

### 2.1. Materials

IPDI was supplied by Covestro AG (Leverkusen, Germany) with the commercial name of Desmodur® I. Dichloromethane (>99.8%, HPLC grade) was obtained from Fisher Chemical (Porto Salvo, Portugal) and used without further purification. PBAT (PBAT A400; M_w_ = 98,000 g/mol) was supplied by Kingfa Sci. & Tech. Co., Ltd. (Guangzhou, China). The emulsifier poly(vinyl alcohol) (PVA) (98−99% hydrolyzed, 57,000–67,000 Da) was obtained from Alfa Aesar (Haverhill, MA, USA).

### 2.2. Microcapsules (MCs) Production

**Scheme 1:**
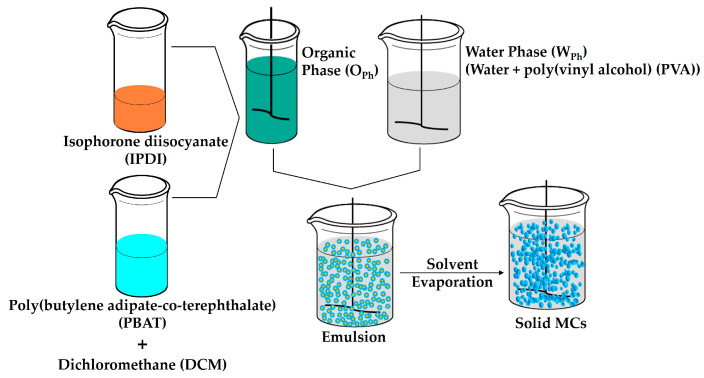
Schematic illustration for the adopted methodology.

The PBAT MCs were produced by organic/aqueous (oil-in-water, O/W) emulsions, combined with solvent diffusion and evaporation method as depicted in Scheme 1. 

The parameters indicated in Table 1 are related to the emulsion system used to produce the reference MCs (R-MCs). The organic phase was prepared after 3.25 g of PBAT was dissolved in 25 g (18.8 mL) of dichloromethane and magnetically stirred until it formed a clear solution. Then, the polymer’s solution was added to 6.8 g (31 mmol) of IPDI and mixed at 500 rpm for 5 min. The IPDI and the polymeric solution correspond to 7.8 wt% and 33 wt% of the final emulsion system, respectively. The organic phase (O_Ph_) was dispersed in the water phase (W_Ph_) under mechanical stirring (750 rpm), at room temperature. The water phase was composed of an aqueous solution with 3 wt% of PVA (1.5 g). The emulsion system was left under mechanical stirring for 3 h to produce MCs with enough resistance to tolerate the pressure applied during the filtration. The MCs were filtrated, washed with water (400 mL), and left to dry overnight. In addition, 50% of the MCs were stored in a desiccator at room temperature (20–25 °C) and the other 50% of the MCs were left in an atmosphere of 60% of relative humidity (RH) at room temperature (20–25 °C).

The production yield was calculated by the following Equation (1), where “*m*_total_” is the total mass of PBAT and IPDI used for MCs preparation and “*m*_MCs_” is the gross mass of MCs.

(1) $$Yield=\frac{m_{\mathrm{MCs}}}{m_{\mathrm{total}}}100\;(\%)$$

A set of experiments (Table 2) were performed to study the effect of the different parameters on the characteristics of the MCs. For instance, to study the parameter “amount of PVA” 6 samples were prepared with different PVA weights in the aqueous phase, while the other conditions did not vary. The production time and the W_Ph_ weight was kept constant in all studies. It should be stressed that for each MC type disclosed in Table 2, three identical and independent syntheses were performed. The IPDI loading and the MCs size and morphology were assessed for all samples.

### 2.3. Microcapsules’ (MCs) Characterization

#### 2.3.1. Optical Microscopy and Scanning Electron Microscopy (SEM)

The MCs formation, maturation, and size (bigger MCs) were assessed by optical microscopy (Kruss MSZ 5600 optical microscope (Hamburg, Germany)). 

The morphology, surface roughness, porosity, and size (smaller MCs) were evaluated through scanning electron microscopy (SEM), with a Phenom ProX G6 benchtop SEM (ThermoScientific, Waltham, MA, USA). The samples were immobilized in a sample holder by using a conductive double-sided adhesive tape and coated with a 15 nm layer of a conductive Au/Pd thin film, through sputtering, by using a turbomolecular pumped sputter coater from Quorum Technologies, model Q150T ES (Lewes Road, Laughton, UK). 

The size measurement of the MCs was evaluated by using photomicrographs obtained through optical microscopy and/or by SEM, employing the Fiji software (ImageJ/Fiji 1.53k) [44]. The SEM images were obtained with a magnification of 420× (scale bar = 150 µm), 1200× (scale bar = 100 µm), 1650× (scale bar = 40 µm), and 5000× (scale bar = 15 µm). The optical microscopy images were obtained with a magnification of 20× (scale bar = 300 µm) and 40× (scale bar = 100 µm).

The MCs sizes are represented by the mean values (differential size distribution) and D_10_, D_50_ (median), and D_90_ values which were calculated through the intercepts for 10%, 50%, and 90% of the cumulative distribution. The span ((D_90_ − D_10_)/D_50_) is a dimensionless measure of width but it introduces a considerable error when the distributions have several mode values (peaks) [45]. Since some of the size histograms present a random distribution, in this work, we did not calculate the span. Instead, we used D_90_ − D_10_ to analyze the width (range of particle size distribution). In the Supplementary Information (Tables S1–S5) the quartiles 25% (Q1) and 75% (Q3) or maximum and minimum values are also available. 

#### 2.3.2. Gel Permeation Chromatography (GPC)

GPC analysis was used to verify the molecular weight (MW) of the PBAT (raw and MCs). The analyses were performed on a Jasco system (Tokyo, Japan) equipped with a refractive index detector of model RI4030 and a UV−vis detector of model UV-4070 (set at 254 nm), equipped with a precolumn SDV and a SDV linear column (PSS SDV analytical linear S). The calibration curve was established by using polystyrene standards. An isocratic method was followed by using chloroform as the mobile phase at a flow rate of 1.5 mL/min at 40 °C. The samples were prepared at a concentration of 1 g/L in chloroform.

#### 2.3.3. Fourier Transform Infrared Spectroscopy (FTIR)

FTIR was used to confirm the encapsulation of IPDI and to evaluate the formation of PU/PUa moieties. With that intent, it was used a Spectrum Two FT-IR spectrometer from PerkinElmer (Waltham, MA, USA) equipped with a UATR Two accessory. The spectra were obtained at 4 cm^−1^ resolution and 8 scans of data accumulation.

#### 2.3.4. Proton Nuclear Magnetic Resonance (^1^H NMR)

^1^H NMR spectra were acquired in a Bruker Avance III 400 spectrometer (Karlsruhe, Germany) with CDCl_3_ as solvent. For IPDI quantification, 4-chloro-3-methylphenol was used as an internal standard.

#### 2.3.5. Thermogravimetric Analysis (TGA) and Derivative Thermogravimetry (DTG)

The TGA thermograms were achieved using a Hitachi STA 7200 thermal analysis system (Ibaraki, Japan) under a controlled nitrogen atmosphere with a flow of 200 mL/min, at a temperature increase rate of 10 °C/min, in the range of 35−600 °C. The thermal stability of the microcapsules was characterized by measuring the weight loss with the temperature increase.

#### 2.3.6. Viscosity Measurements

The viscosity measurements of the samples were performed in a Modular Compact Rheometer (MCR) Series – MCR 92 from Anton Paar (Graz, Austria) with a maximum torque of 125 mNm and a minimum torque rotation/oscillation of 1 μNm. Flow tests were performed with a cone-and-plate system composed of a measuring cone CP50-1 with a diameter of 50 mm and an angle of 1°. Disposable dishes EMS/CTD 600, from Anton Paar (Graz, Austria), with 54 mm diameter were also used. A gap of 1.01 mm between the measuring cone and the Aluminum blend disposable dish was maintained. A total of 0.5 mL–1.5 mL of the sample was used for the measurements, depending on the viscosity sample. A rest time of 25 s was used for stabilizing the sample before the essay. A shear rate ranging from 1 to 1000 s^−1^ was applied. From the 21 points taken, the software interpolated the viscosity value for 50 s^−1^ after accomplishing the flow curves. Each test was performed three times and the temperature was maintained at 25 °C.

#### 2.3.7. Statistical Analysis

The MCs production and the viscosity measurements were performed in triplicate. The obtained data are expressed as mean ± standard deviation values. The size of the MCs was evaluated in a sample of at least 250 MCs (“N Analysis”), unless indicated otherwise. Statistical analysis was determined by nonparametric analysis (Kruskal–Wallis test and Dwass–Steel–Critchlow–Fligner test) using Jamovi software, version 2.3.21 [46].

## 3. Results and Discussion

### 3.1. MCs (R-MCs) Production and Characterization

#### 3.1.1. Encapsulation Process 

**Figure 1:**
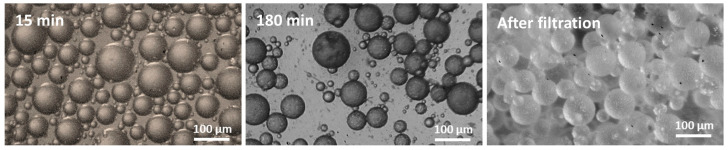
Optical microscopy photographs taken during the production of R-MCs and after the filtration process.

For the encapsulation process, we choose dichloromethane (DCM) as an organic solvent because of its low boiling point and low miscibility with water. Additionally, its high saturated vapor pressure assures a high solvent evaporation rate, which shortens the production time. During the process of solvent evaporation, the DCM gradually diffuses into the continuous phase and evaporates. This feature causes small polymer-rich domains that change the organic droplet from a one-phase (O_Ph_) to two-phase particle (O_Ph1_/O_Ph2_). Due to interfacial tension interactions between the core, polymer, and aqueous phase, the polymer-rich domains migrate to the surface of the organic droplets where they merge and spread to wrap the original oil droplet [47,48]. The entire process was followed by optical microscopy to check the evolution of the emulsion and the robustness of the MCs’ shell. After 180 min, the MCs offered enough resistance to the pressure applied during the filtration procedure (Figure 1). 

As shown in Figure 1, the R-MCs are spherical, loose, and homogeneous. With small exceptions, the MCs produced under different conditions (Table 2) exhibit similar characteristics. Due to the intrinsic properties of PBAT (glass transition temperature of about −30 °C and Young’s modulus above 400 MPa), the MCs are rubbery and tough. Almost all syntheses produced MCs with an efficiency above 70% and most of the losses are due to some accumulation at the walls of the reaction vessel and mixer. GPC analyses of the PBAT and the MC’s shell indicate that the molecular weight and molecular weight distribution did not change with the microencapsulation process (Figure S1).

#### 3.1.2. Characterization of R-MCs and IPDI Load

**Figure 2:**
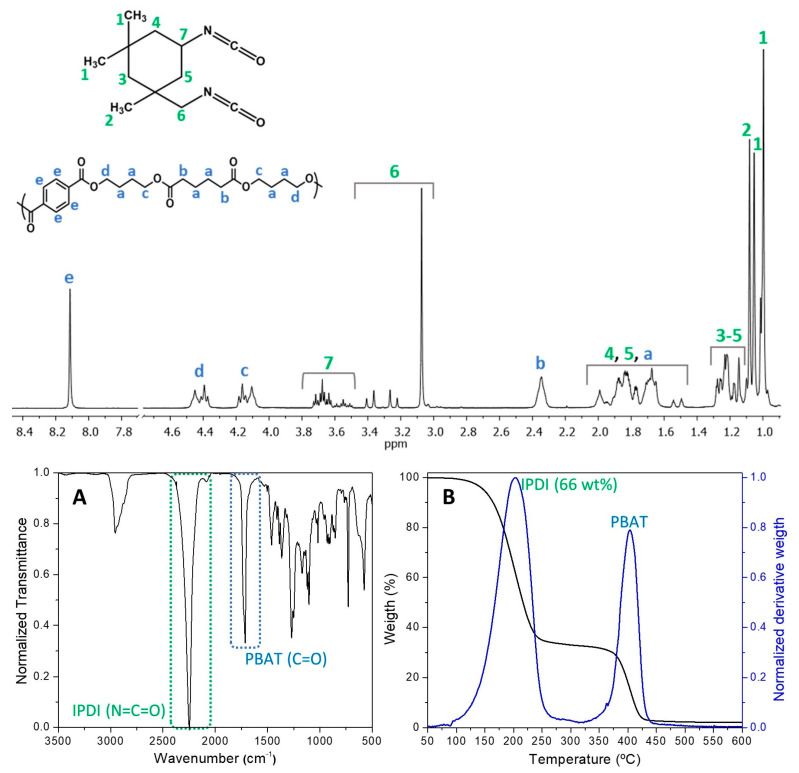
^1^H NMR spectrum of R-MCs (up), FTIR spectrum (**A**) and thermogram (**B**) of R-MCs.

The R-MCs were characterized by ^1^H NMR, FTIR, and TGA (Figure 2). If compared with the IPDI and PBAT individual spectra (Figure S2), it is possible to conclude that the ^1^H NMR spectrum of the R-MCs is a perfect merge of both spectra. In Figure 2, the peaks are assigned according to their structure, and no unexpected peaks were found. The signals assigned as “6” and “7” correspond to the two H protons in –CH_2_NCO (primary NCO) and the H proton in –CHNCO (secondary NCO), respectively. The assignments include both *cis* and *trans* isomers and, in both cases, the ratio between the isomers is 100:31. 

PUa formation, by IPDI reaction with water, affects the signals of the hydrogen protons, especially the protons directly correlated with the isocyanate group (Figure S3). Since signals “6” and “7” preserve their multiplicity, shape, and chemical shifts we discarded the occurrence of a relevant polymerization.

The FTIR analysis aims to detect the presence of isocyanate groups, evaluate the degree of polymerization, and access the MCs’ shell composition. In Figure 2A, the intense band at 2254 cm^−1^ attributed to N=C=O stretching vibration, and the carbonyl group (C=O) stretching vibrational band at 1718 cm^−1^, from PBAT, are very well defined. The broad band between 2774 cm^−1^ and 3036 cm^−1^ is ascribed to C–H stretching from aliphatic and aromatic segments. The signals at 1271 cm^−1^ and 1104 cm^−1^ are assigned to stretching vibration of the C–O–C in the ester linkage connected to benzene rings, and the signals at 1120 cm^−1^ and 1168 cm^−1^ are for the same sort of bonding but for the ester linkage connected to the aliphatic acid structure instead [49]. The sharp peak at 731 cm^−1^ is due to the out-plane bending mode of =C–H in the benzene ring.

TGA and ^1^H NMR assessments were used to quantify the encapsulated IPDI (wt%) immediately after the production. For ^1^H NMR quantification, 4-chloro-3-methylphenol was used as internal standard (Figure S4) and the proton in –CHNCO from IPDI was chosen to do the correlation. Figure 2B depicts the thermograms of the R-MCs and the corresponding derivative curves (DTG). IPDI presents a thermal event associated with its evaporation starting around 90 °C and having a maximum degradation temperature (T_max_) at 203 °C, that was used to estimate the IPDI loading. PBAT shows a single degradation event, with T_max_ value around 408 °C (Figure S5). 

R-MCs presented a thermogram with a combined effect of IPDI’s mass loss event and PCL’s mass loss event. Nevertheless, an extra band was verified at around 315 °C. This new thermal event represents only 1 wt% of the R-MCs and indicates the residual conversion of isocyanate into PUa, by reaction with moisture. The R-MCs degrade almost completely with a char yield of 2.1% at 600 °C, under an inert atmosphere, which is consistent with the literature data [50]. The IPDI content obtained by NMR was 68 wt% and by TGA was 66 wt%, which are very close values and indicate a high encapsulation efficiency.

#### 3.1.3. Stability Tests

**Figure 3:**
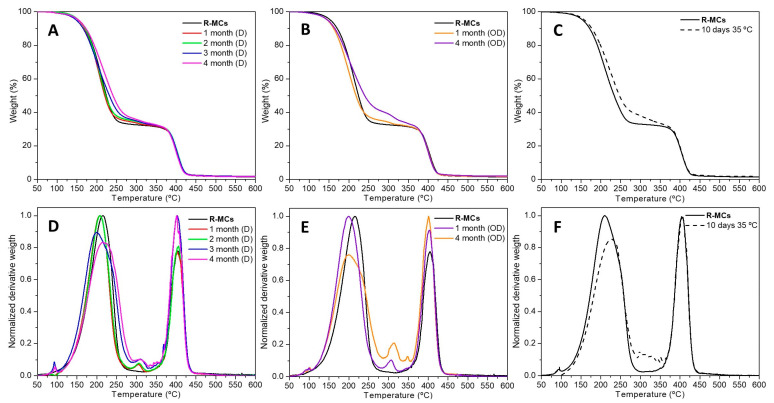
Thermograms and derivative curves of R-MCs over time stored in a desiccator (**A**,**D**), under normal environmental conditions out of a desiccator (**B**,**E**), and after 10 days at 35 °C (**C**,**F**).

The MCs protection ability was assessed by different studies and the IPDI load was verified by TGA. To assess the stability over time, at room temperature, 50% of the R-MCs were packed in glass vials and stored for 4 months in a desiccator (D), to protect them from atmospheric humidity, and the remaining MCs were stored out of a desiccator (OD) at room temperature and 60% of relative humidity. Additionally, a tube with R-MCs was also stored in an oven at 35 °C for 10 days. Figure 3 displays the thermograms and respective derivative curves. 

Unsurprisingly, the MCs stored in the desiccator (D) present better behavior in terms of stability over time, since they only lost 6% of their initial IPDI load after 4 months. However, the MCs stored out of the desiccator (OD) only lost 11% which is a positive result. We can assume that the PBAT shell protects the encapsulated isocyanate relatively well. The DTG curve of the MCs after 4 months (Figure 3D,E) presents a widening of the band related to the IPDI loss. This feature may be related to the formation of IPDI oligomers. After 10 days at 35 °C, the MCs lost 9% of their IPDI content which suggests that the polymerization of IPDI is catalyzed in moderate temperatures. 

**Figure 4:**
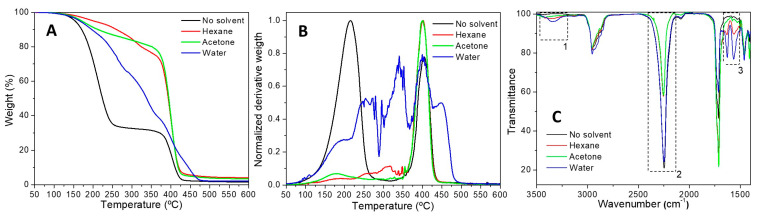
Thermograms (**A**), thermogram derivative curves (**B**) and FTIR spectra (**C**) of **R-MCs** after dispersion in different solvents.

The resistance to solvents was tested by dispersing 10 mg of R-MCs in 5 mL of hexane, acetone, or distilled water for 3 days. As displayed in Figure 4, the IPDI was leached by hexane and acetone. The appearance of the thermal events between 250 °C and 350 °C suggests that a small portion of PUa was formed when the MCs were dispersed in hexane. This is probably due to some moisture outside the MCs and the solution-air interface. The dispersion in acetone does not create a significant amount of PUa. The dispersion in water does not seem to have caused as much leaching as the other organic solvents. On the other hand, it resulted in the formation of a significant amount of PUa, which indicates that some water has entered the MCs. Koh et al. described the existence of two weight loss steps for PU/PUa materials: the first at around 260–350 °C associated with the degradation of soft segments and a second loss at around 360–490 °C due to the hard domains [51]. These two weight loss steps are evident in Figure 4B. 

The polyurea formation or the simple isocyanate leaching can also be noted by FTIR analysis (Figure 4C). Boxes 1 and 3 delimit the bands attributed to N–H bending and stretching, respectively, as well as the band associated with stretching of C=O group (1626 cm^−1^). The decrease in the band assigned to N=C=O bond stretching (2) is more related to the leaching of isocyanate. 

SEM images of the MCs before and after the dispersion in acetone (Figure S6) show that the MCs sustain their shape and were not ruptured or broken. Nevertheless, the surface of the MCs has been slightly modified.

### 3.2. Effect of Process Parameters 

In the solvent evaporation technique, core content, morphology, size, size dispersion and encapsulation efficiency are deeply influenced by processing parameters. We systematically studied the effects of the different parameters to obtain spherical, loose, core-shell MCs with a high IPDI load. The effect of the different parameters (stirring rate, PVA, PBAT, DCM and IPDI amounts) on MCs size, size distribution (Figures S7–S11 and Tables S1–S5), and IPDI loading was object of intense work. The parameter values used for R-MCs production act as references in every study.

#### 3.2.1. Mechanical Stirring Rate

In emulsion systems, the stirring rate is one of the most important parameters to achieve MCs and control their size. It regulates the equilibrium between the shear and interfacial tension forces of the oil droplets [17]. Under high stirring, the shear forces overcome the interfacial tension forces and produce small droplets. However, severe agitation can burst the droplets and incorporate air, resulting in loss of surfactant at the water-air interface. Coalescence phenomena are favored at low mixing rates and contribute to the enlargement of the droplets. It has been reported that there is a linear relationship between stirring rate, mean size, and size distribution [52,53]. 

**Figure 5:**
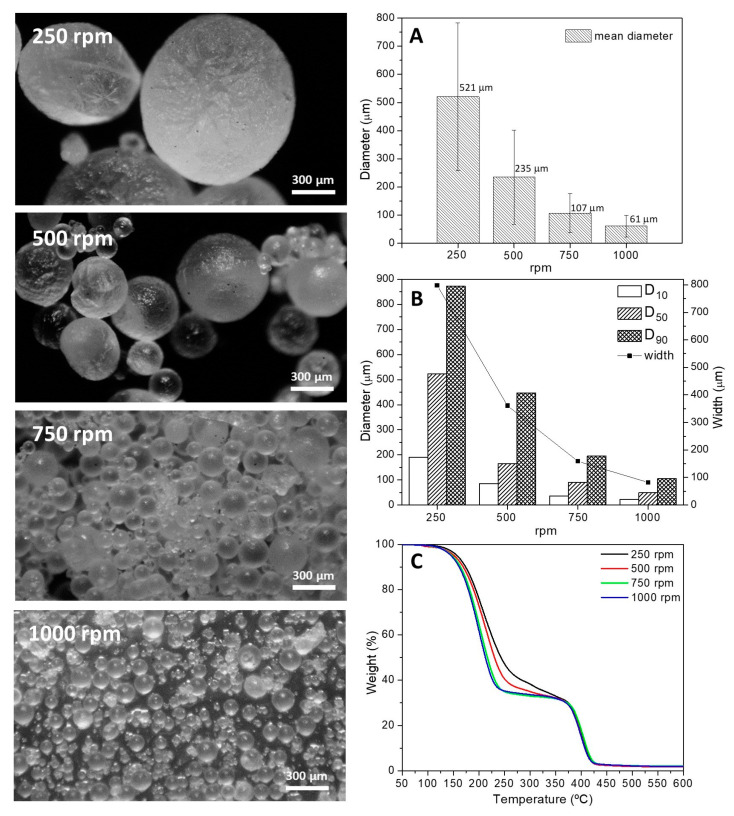
Optical microscopy photograph showing the effect of mechanical stirring rate on MCs size, the effect of stirring rate on the mean diameter (**A**), diameter (D_10_, D_50_, D_90_), and width values from MCs distribution (**B**); thermograms of MCs (**C**).

The results in Figure 5 confirm that the mean size, median size (D_50_), and distribution width decrease with the increase in stirring rate. The size histograms of the MCs (Figure S7) show skewed distributions to the right except for those obtained at 250 rpm which produced a histogram with random distribution. With the increase in agitation rate, large droplets were sheared and dispersed in the continuous phase, and they had no chance to merge. Consequently, the MCs prepared at a higher agitation rate (750 rpm and 1000 rpm) have smaller sizes and the distribution is more uniform (lower width). In these MCs, the difference between the mean and D_50_ is also much smaller. The statistical analysis (Table S1) indicates that there is a significant difference between the D_50_ values.

SEM images (Figure S12) confirm the core-shell typology of the MCs obtained between 500 rpm and 750 rpm, and matrix type for those obtained at 250 rpm. In the core-shell MCs, it is possible to observe that the inner and outer surfaces of the shells are very similar. Despite the differences between MCs, all syntheses produced rounded capsules with IPDI encapsulated. As shown in Figure 5C, the MCs produced at 250 rpm and 500 rpm present a lower content of pristine IPDI than those produced at 750 rpm and 1000 rpm (62 wt% and 64 wt% vs. 66 wt%). This feature is justified by coalescence and aggregation issues. 

#### 3.2.2. Amount of Poly(Vinyl Alcohol) (PVA) 

We used PVA in the water phase as emulsifier to enhance the emulsion stability. The purpose of emulsifiers in a liquid–liquid dispersion is to minimize the effects of interfacial forces between the immiscible liquids by forming a protective layer around the droplets. They are crucial to avoiding droplets’ coalescence and aggregation, providing electrostatic and/or steric repulsion and reducing the interfacial tensions between the two phases. The PVA concentration could have an offsetting effect on MCs’ size since there are reports on the decrease in particle size with the increase in PVA amount, and the opposite as well [21,54,55,56]. Up to reaching the critical aggregation concentration, more PVA ensures better system stabilization against the coalescence of the emulsion and leads to smaller MCs. However, when its concentration reaches a high level, the risk of depletion flocculation occurrence or other destabilizing factors is considerable [57].

**Figure 6:**
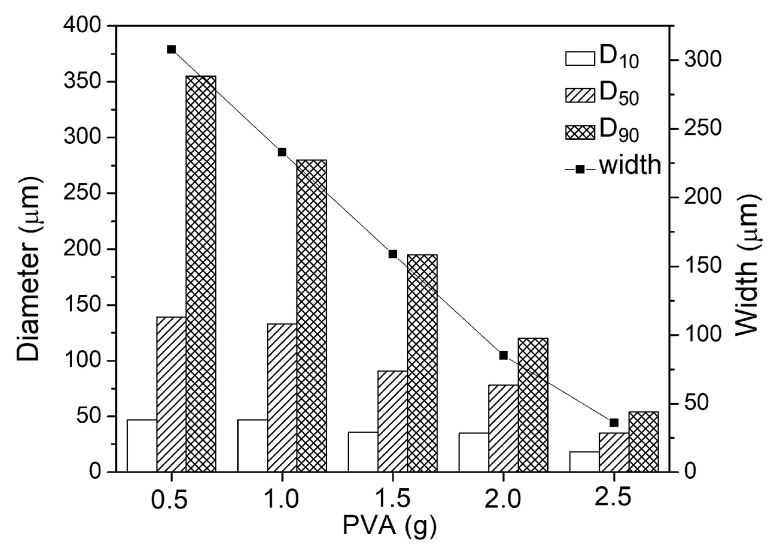
Effect of PVA amount on diameter (D_10_, D_50_, D_90_) and width values from MCs distribution.

In this study, five samples were prepared with different amounts of PVA (0.5–2.5 g). The aqueous phase was always set at 50 g, therefore, the PVA concentration ranged from 1 wt% to 5 wt%. As stated in Table 3 and Figure 6, the mean and median diameter values decrease from 171 µm to 37 µm as the PVA amount increases from 0.5 g to 2.5 g. The distribution width decreases as well. A higher concentration of PVA reduced the interfacial tension and ensured better system stabilization against the coalescence of the emulsion. The size histograms of the MCs (Figure S8) display a right-skewed distribution, with a more prominent tail in the distributions of MCs obtained with less PVA. The D_50_ values are significantly different except for the 1.0–0.5 g comparison (Table S2). A test without PVA formed an unstable emulsion that made it impossible to produce MCs.

**Figure 7:**
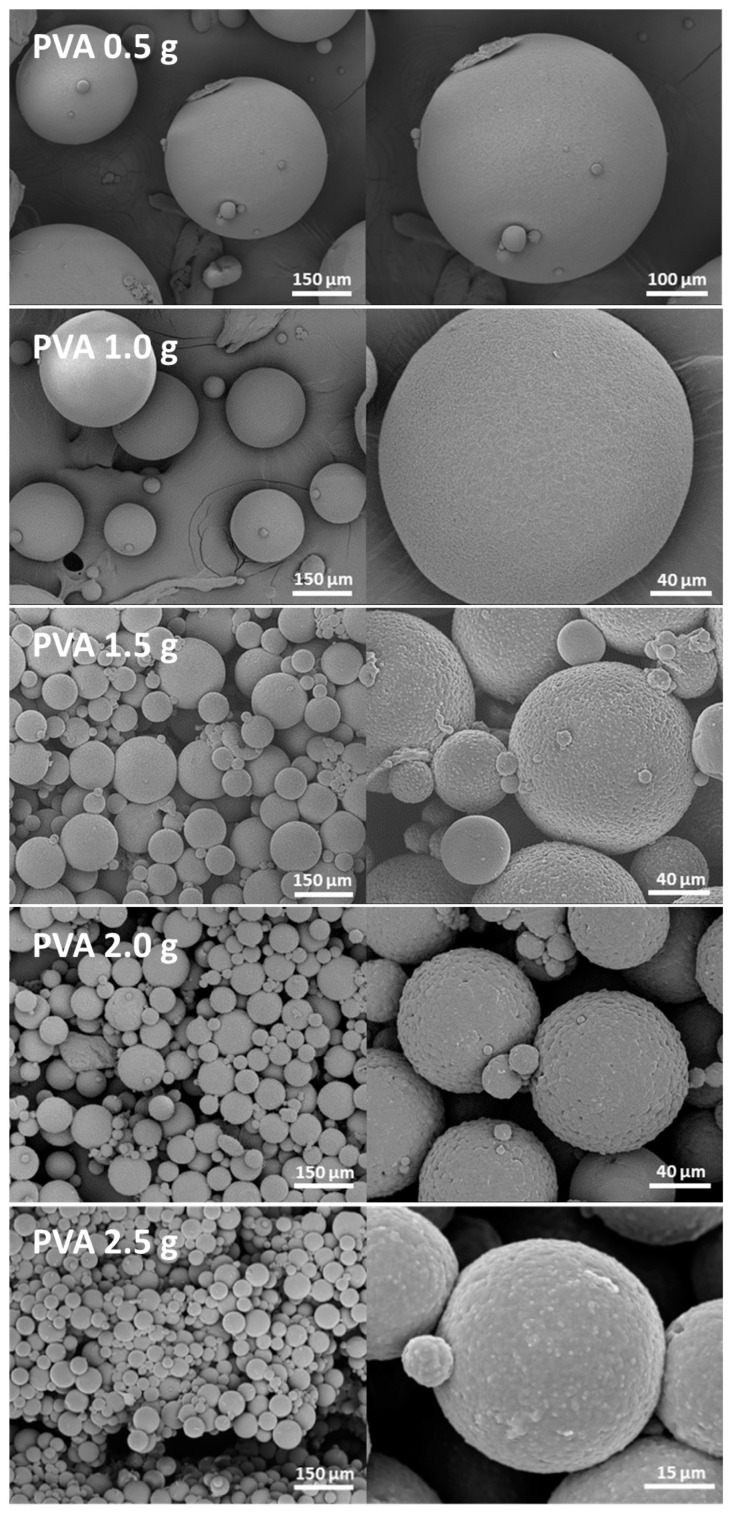
SEM images of MCs obtained with different PVA amounts.

Through SEM (Figure 7), we conclude that all syntheses produced MCs with a regular rounded form, without holes or cracks. Nevertheless, less PVA led to more debris or shapeless structures formation (Figure S13), which is justified by the formation of more unstable emulsions. It was also possible to verify that the MCs (PVA 0.5–1.5 g) have a core-shell typology. The shell is very thin and there are no big differences between the outer and inner surfaces. Due to the small size of the MCs obtained with 2 g and 2.5 g and the high elongation at break and toughness of PBAT, it was not possible to verify if those MCs have core-shell typology.

The increase in PVA concentration did not only lead to smaller MCs but also affected the surface morphology of capsules. The biggest MCs (obtained with 0.5 g and 1 g of PVA) exhibit smoother surfaces than those obtained with 1.5 g. This trend is quite clear from 1 g to 1.5 g or from 1.5 g to 2 g. We did not find a negative effect at higher amounts of PVA, which leads us to believe that the water surface tension was not exceeded. The smaller MCs were more difficult to separate, i.e., more agglomerated, since their small size triggers electrostatic attraction and surface forces between them. Nevertheless, some MCs are physically attached which could result from an excessive PVA amount.

The IPDI content (Figure S14) was essentially the same in all cases (66–67%), which certifies the robustness of this method and proves that the IPDI was very well protected by the PBAT shell. 

#### 3.2.3. Amount of Poly(Butylene Adipate-*Co*-Terephthalate) (PBAT)

**Figure 8:**
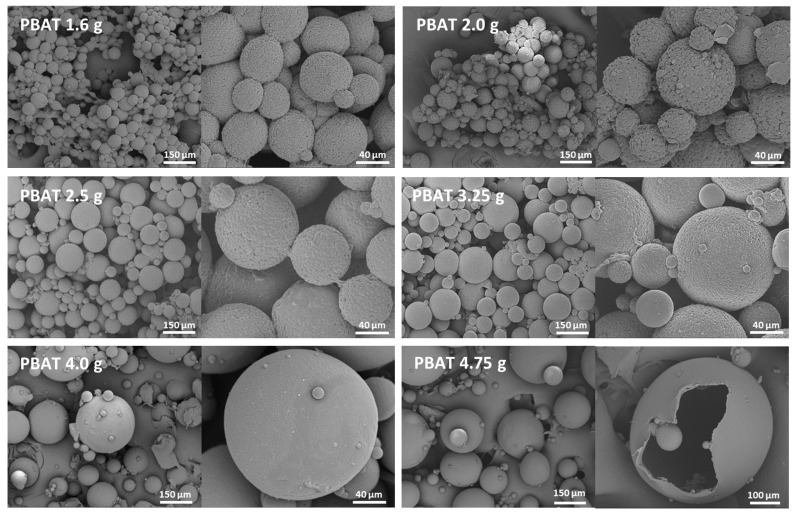
SEM images of MCs obtained with different PBAT amounts.

**Figure 9:**
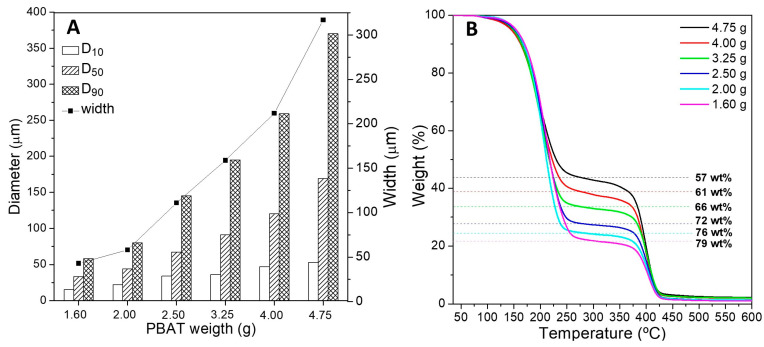
Diameter length (D_10_, D_50_, D_90_) and width values from MCs obtained with different PBAT amounts (**A**). Thermograms from MCs obtained with different PBAT amounts (**B**).

The polymer’s amount is an important factor that impacts the particle’s characteristics such as size, distribution, encapsulation efficiency, or surface morphology. Herein, the polymer amount was varied from 1.60 g to 4.75 g, while the other conditions were kept constant. The results obtained (Table 4, Figure 8 and Figure 9) imply that an increase in the polymer’s amount in a fixed volume of organic solvent (18.8 mL), i.e., higher concentration, leads to a gradual increase in MCs’ diameter and distribution width. This might be due to the more viscous medium since the size of MCs depends on the net shear stress available for droplet breakdown, and viscous forces oppose the shear stresses in the organic phase.

The mean and D_50_ size diameter values are very close when the amount of PBAT is low (1.6 g and 2 g), and all D_50_ values are statistically different (Table S3). With the increase in PBAT content, the difference goes up (19–26 µm) and the asymmetries in the size distribution histograms (Figure S9) are more marked.

The MCs with reduced amounts of PBAT (1.6 g to 2.5 g) are the smallest, but after filtration and drying, they tend to be attached to each other. This clustering could result from excessive PVA or due to the surface tension of water on MCs during the drying. At the microscope, it was possible to see well-dispersed MCs without clumps (Figure S15). 

The dosage of more PBAT should raise the number of MCs [56]. However, it also increases the viscosity and reduces the stabilizing efficiency of PVA, which leads to the coalescence of the droplets. Smaller droplets have a bigger surface area and more PVA concentration at the surface, which could impact the surface morphology. The bigger MCs have smoother surfaces than the smaller MCs. As can be stated by analyzing the broken MC presented in Figure 8 or Figure S16, the high content in PBAT did not change the MCs typology (remained core-shell). The shell is still very thin (less than 0.5 µm), and the inner surface is very similar to the outer surface.

As depicted in Figure 9B, the IPDI load significantly varies from 57 wt% to 79 wt%. The low amount of PBAT produced MCs with higher loadings. A few works present MCs with isocyanate load (core content) above 70 wt% [38,58,59,60,61,62,63,64,65]. However, it should be noted that the term “core content” often includes solvents, oligomers, and isocyanate prepolymers, which means that the encapsulated material is not all composed of compounds with free -NCO groups [8], as desired. As far as we know, the loadings of 72 wt%, 76 wt%, or 79 wt% of pristine IPDI, achieved in this work, are among the highest published to date. The syntheses involving the larger amounts of PBAT (4 g and 4.75 g) produced MCs with 2–3 wt% of PUa. The higher amount of char (between 500 °C and 600 °C) is also related to the higher percentage of PBAT.

#### 3.2.4. Amount of Dichloromethane (DCM)

The quantity of organic solvent (DCM in our case) has the same effect on PBAT’s concentration as using more or less PBAT and keeping the volume of solvent. However, when we change the volume of the organic solvent, the total volume of the organic phase changes as well, which affects emulsion viscosity and polymer precipitation rate. 

**Figure 10:**
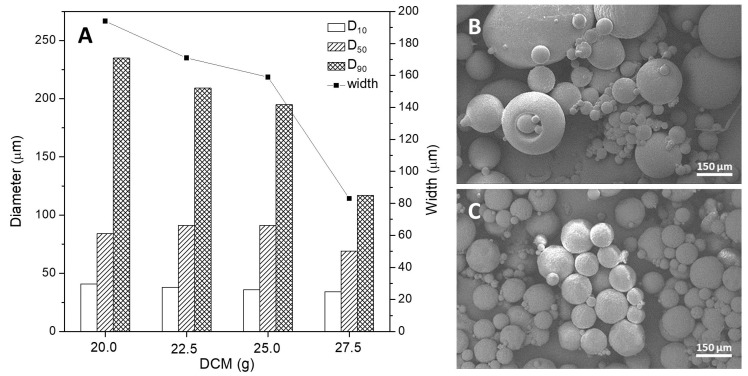
Diameter length (D_10_, D_50_, D_90_) and width values from MCs obtained with different DCM amounts (**A**), SEM images of MCs obtained with 20.0 g (**B**) and 27.5 g (**C**) of DCM.

More DCM (lower PBAT concentration) leads to smaller MCs, but the D_50_ differences between the syntheses using 20 g, 22.5 g, and 25 g are not significant (Table 5 and Table S5). The MCs obtained with less DCM presented wider size distributions (Figure 10 and Figure S10), which made the median and mean have noticeable discrepancies and showed some signs of MCs’ malformation (Figure 10). In addition, the MCs produced with smaller amounts of DCM exhibit an IPDI encapsulation of 64%, while the others presented a slightly higher encapsulation (Table 5 and Figure S17).

#### 3.2.5. Amount of Isophorone Diisocyanate (IPDI)

The compound to be encapsulated can greatly impact the quality of the emulsion, the morphology, and the properties of the final MCs. When we try to encapsulate isocyanate derivatives, namely specific isocyanate pre-polymers, or oligomers, this is even more important since they are very reactive. Therefore, it is essential to find a balance to encapsulate as much as possible, but at the same time provide as much protection as possible.

**Figure 11:**
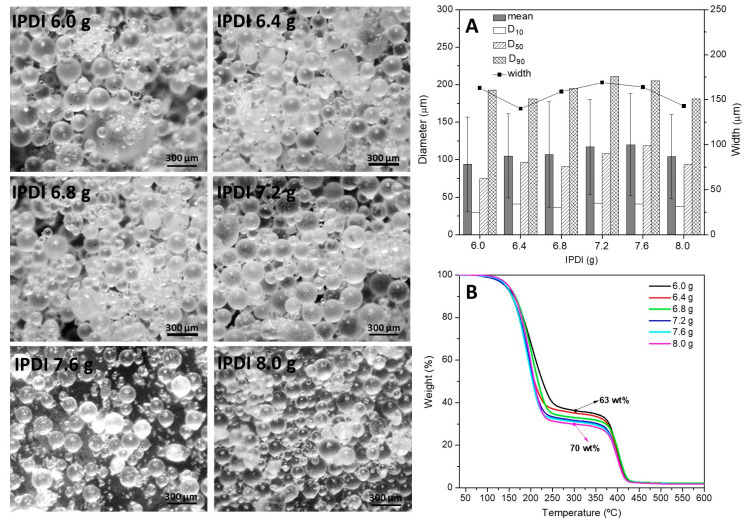
Optical microscopy photograph from the MCs produced with different amounts of IPDI; Diameter length and width values of the MCs (**A**), thermograms from MCs obtained with different IPDI amounts (**B**).

As shown in Figure 11, we varied the amount of IPDI between 6 g and 8 g, keeping all other parameters unchanged. The diameter was slightly enlarged with the increase of IPDI content (from 6 g to 7.20 g), and it decreased when 8 g was used. IPDI is a liquid isocyanate with low viscosity (10 cP). Consequently, an increasing amount of IPDI will decrease the viscosity of the system (Table S6) and facilitate droplet breakdown. On the other hand, the increase of a liquid core content can lead to a ballooning effect, as the size of the MCs grows to a certain point where the droplets burst. Thus, the increasing or decreasing in IPDI content has two opposite effects on the emulsion. As presented in Figure 11 (A), we did not find a trend regarding size distribution, and the pair-wise comparisons (Table S5) suggest a few not significant differences between D_50_ values (6.4–6.8 g, 6.4–7.2 g, 6.4–8.0 g, 7.2–7.6 g, and 6.8–8 g). The syntheses that produced MCs with more uniform sizes were those with 6.40 g of IPDI. 

The thermograms (Figure 11B) show a regular increase of encapsulated IPDI with the increase of IPDI weight per synthesis. None of the examples show the formation of significant amounts of PUa, showing that within these values the emulsion is stable. The fact that the char yield at 600 °C is similar for all examples also indicates that the amount of PBAT is analogous for all.

## 4. Conclusions

In this study, we demonstrate the encapsulation of an isocyanate derivative (IPDI) using PBAT as biodegradable shell material. These novel MCs present a regular surface, without holes or cracks, a very thin shell, and high isocyanate loading, up to 79 wt%. We also demonstrate that the PBAT shell protects IPDI from air moisture, a critical feature for a long shelf-life.

In addition, we systematically studied the impact of the production parameters on MCs size, morphology, and IPDI loading. The increase of different parameters such as stirring rate (1000 rpm), amount of PVA (2.5 g), or organic solvent (27.5 g) leads to smaller MCs. On the contrary, the increase of PBAT amount (4.75 g) leads to larger MCs. In this way, we can produce rounded PBAT MCs with tailored mean diameters between 36 µm and 521 µm (median 34–523 µm). Throughout this study, the IPDI loading was obtained as theoretically expected, and apart from a few extreme variations, the polymerization extension of IPDI, in the form of PUa, represented only 1 or 2 wt% of the MCs. This study provides valuable information for future works since it presents the first report on PBAT MCs loaded with isocyanate, and it explains several correlations that greatly impact the size and quality of these MCs.

## Figures and Tables

**Table 1:** Parameters to produce reference MCs (R-MCs).

Stirring Speed (rpm)	PVA (g)	m W_Ph_ (g)	m PBAT (g)	m DCM (g)	m IPDI (g)	m O_Ph_ (g)
750	1.5	50	3.25	25	6.8	35.05

**Table 2:** The different sets of experiments. The parameters to produce R-MCs (750 rpm; 1.5 g of PVA, 3.25 g of PBAT, 25 g of DCM, and 6.80 g of IPDI) act as a reference in each study.

Parameter	Stirring Speed (rpm)	PVA (g)	m PBAT (g)	m DCM (g)	m IPDI (g)	Yield (%)
Stirring speed	250	1.5	3.25	25	6.80	86.7 ± 2.1
	500	1.5	3.25	25	6.80	85.7 ± 2.1
	750	1.5	3.25	25	6.80	79.3 ± 3.7
	1000	1.5	3.25	25	6.80	71.7 ± 1.7
Amount of PVA	750	0	3.25	25	6.80	0 ± 0
	750	0.5	3.25	25	6.80	78.7 ± 3.7
	750	1	3.25	25	6.80	78.0 ± 3.7
	750	1.5	3.25	25	6.80	79.3 ± 3.7
	750	2	3.25	25	6.80	80.6 ± 0.5
	750	2.5	3.25	25	6.80	78.3 ± 0.5
Amount of PBAT	750	1.5	1.60	25	6.80	52.0 ± 9.6
	750	1.5	2.00	25	6.80	65.7 ± 3.9
	750	1.5	2.50	25	6.80	69.7 ± 0.5
	750	1.5	3.25	25	6.80	79.3 ± 3.7
	750	1.5	4.00	25	6.80	82.3 ± 1.9
	750	1.5	4.75	25	6.80	76.7 ± 0.5
Amount of DCM	750	1.5	3.25	20.0	6.80	75.7 ± 1.7
	750	1.5	3.25	22.5	6.80	77.7 ± 0.5
	750	1.5	3.25	25.0	6.80	79.3 ± 3.7
	750	1.5	3.25	27.5	6.80	85.7 ± 1.2
Amount of IPDI	750	1.5	3.25	25	6.00	83.7 ± 0.5
	750	1.5	3.25	25	6.40	82.7 ± 2.5
	750	1.5	3.25	25	6.80	79.3 ± 3.7
	750	1.5	3.25	25	7.20	70.3 ± 5.3
	750	1.5	3.25	25	7.60	77.0 ± 1.6
	750	1.5	3.25	25	8.00	83.7 ± 0.9

**Table 3:** Effect of PVA amount on viscosity and mean diameter length.

PVA (g)	PVA Solution Viscosity (cP)	Emulsion Viscosity (cP)	Mean Size (µm)
0.5	2.290 ± 0.08	25.76 ± 1.50	171 ± 121
1	4.840 ± 0.060	31.91 ± 0.64	149 ± 89
1.5	10.60 ± 0.15	61.35 ± 0.71	107 ± 70
2	24.08 ± 1.09	79.43 ± 1.24	81 ± 30
2.5	36.11 ± 0.50	99.48 ± 1.21	37 ± 14

**Table 4:** Effect of PBAT amount on viscosity and MCs mean diameter length.

PBAT (g)	PBAT Solution Viscosity (cP)	Emulsion Viscosity (cP)	Mean Size (µm)
1.60	61.73 ± 0.09	39.31 ± 0.23	36 ± 16
2.00	85.81 ± 0.38	45.62 ± 0.59	49 ± 22
2.50	114.5 ± 0.38	51.40 ± 0.20	86 ± 53
3.25	208.4 ± 0.69	61.35 ± 0.71	107 ± 70
4.00	321.25 ± 0.48	81.88 ± 0.36	143 ± 99
4.75	461.61 ± 0.38	103.4 ± 1.8	195 ± 125

**Table 5:** Effect of DCM amount on viscosity, MCs size, and IPDI loading.

DCM (g)	PBAT Solution Viscosity (cP)	Emulsion Viscosity (cP)	Mean Size (µm)	IPDI (wt%)
20.0	327.0 ± 0.2	123.5 ± 1.3	112 ± 88	64
22.5	259.0 ± 2.3	85.17 ± 0.89	109 ± 71	64
25.0	208.4 ± 0.7	61.35 ± 0.71	107 ± 70	66
27.5	159.3 ± 0.1	47.25 ± 0.22	74 ± 32	66

## Data Availability

Data is contained within the article and Supplementary Material.

## Supplementary Materials

The following supporting information can be downloaded at https://www.mdpi.com/article/10.3390/polym15030665/s1, Figure S1: The GPC analysis performed for samples of PBAT, and MCs dissolved in chloroform; Figure S2: 1H NMR spectra of PBAT (up) and IPDI (down), in CDCl3; Figure S3: 1H NMR spectra of R-MCs and R-MCs with significant weight percentage of PUa, in CDCl3; Figure S4: 1H NMR spectra of R-MCs (16.5 mg) + 4-chloro-3-methylphenol (7.5 mg), in CDCl3.; Figure S5: Thermogram and DTG of IPDI and PBAT; Figure S6: SEM images of the MCs before and after the dispersion in acetone; Figure S7: Histogram of the particle size distribution from the MCs obtained under different mixing rates; Table S1: Set of statistical analysis tables regarding particle size distribution obtained under different mixing rates; Figure S8: Histogram of the particle size distribution from the MCs obtained with different PVA amounts; Table S2: Set of statistical analysis tables regarding particle size distribution obtained with different PVA amounts; Figure S9: Histogram of the particle size distribution from the MCs obtained with different PBAT amounts; Table S3: Set of statistical analysis tables regarding particle size distribution obtained with different PBAT amounts; Figure S10: Histogram of the particle size distribution from the MCs obtained with different DCM amounts; Table S4: Set of statistical analysis tables regarding particle size distribution obtained with different PBAT amounts; Figure S11: Histogram of the particle size distribution from the MCs obtained with different IPDI amounts; Table S5: Set of statistical analysis tables regarding particle size distribution obtained with different DCM amounts; Figure S12: Optical microscopy photograph (250 rpm) and SEM images of the MCs obtained under different mixing rates; Figure S13: SEM images of the MCs obtained with 0.5, 1 and 1.5 g of PVA; Figure S14: TG analysis from the MCs obtained with different PVA amounts; Figure S15: Optical microscopy photograph from the MCs obtained with 2 g of PBAT; Figure S16: SEM images of MCs obtained with different PBAT amounts; Figure S17: TG analysis from the MCs obtained with different DCM amounts; Table S6: Variation of the viscosity of the emulsions with the increase in IPDI.
