# Supplementary material for: Biodegradable Microcapsules of Poly(Butylene Adipate-co-Terephthalate) (PBAT) as Isocyanate Carriers and the Effect of the Process Parameters
Source: Polymers (Basel). 2023 Jan 28;15(3):665. doi: 10.3390/polym15030665 (PMC9921966; doi:10.3390/polym15030665)
Supplement: Supplementary file 1 [file polymers-15-00665-s001.zip › polymers-2172211-supplementary.pdf]

Biodegradable microcapsules of poly(butylene adipate-co-terephthalate) (PBAT) as isocyanate carriers and the effect of the process parameters

António Aguiar [1,2,*], António Mariquito [1], Diogo Gonçalves [1], Isabel Pinho [2] and Ana C. Marques [1,*]

[1] CERENA, DEQ, Instituto Superior Técnico, Universidade de Lisboa, Avenida Rovisco Pais, 1049-001 Lisboa, Portugal;
[2] CIPADE, Av. Primeiro de Maio 121, São João da Madeira, Portugal;
* Correspondence: antonio.luis.aguiar@tecnico.ulisboa.pt (AA); ana.marques@tecnico.ulisboa.pt (A.C.M)

Contents

Figure S1. The GPC analysis performed for samples of PBAT, and MCs dissolved in chloroform 2

Figure S2. ^{1}H NMR spectra of PBAT (up) and IPDI (down), in $CDCl_3$ 2

Figure S3. ^{1}H NMR spectra of **R-MCs** and R-MCs with significant weight percentage of PUa, in $CDCl_3$. 3

Figure S4. ^{1}H NMR spectra of **R-MCs** (16.5 mg) + 4-chloro-3-methylphenol (7.5 mg), in $CDCl_3$. 3

Figure S5. Thermogram and DTG of IPDI and PBAT... 4

Figure S6. SEM images of the MCs before and after the dispersion in acetone. 4

Figure S7. Histogram of the particle size distribution from the MCs obtained under different mixing rates. 5

Table S1. Set of statistical analysis tables regarding particle size distribution obtained under different mixing rates... 5

Figure S8. Histogram of the particle size distribution from the MCs obtained with different PVA amounts. 6

Table S2. Set of statistical analysis tables regarding particle size distribution obtained with different PVA amounts. .. 7

Figure S9. Histogram of the particle size distribution from the MCs obtained with different PBAT amounts. 8

Table S3. Set of statistical analysis tables regarding particle size distribution obtained with different PBAT amounts. 9

Figure S10. Histogram of the particle size distribution from the MCs obtained with different DCM amounts............ 10

Table S4. Set of statistical analysis tables regarding particle size distribution obtained with different DCM amounts. 10

Figure S11. Histogram of the particle size distribution from the MCs obtained with different IPDI amounts. 11

Table S5. Set of statistical analysis tables regarding particle size distribution obtained with different DCM amounts. 12

Figure S12. Optical microscopy photograph (250 rpm) and SEM images of the MCs obtained obtained under different mixing rates. 13

Figure S13. Sem images of the MCs obtained obtained with 0.5, 1 and 1.5 g of PVA 14

Figure S14. TG analysis from the MCs obtained with different PVA amounts. 14

Figure S15. Optical microscopy photograph from the MCs obtained with 2g of PBAT.. 15

Figure S16. SEM images of MCs obtained with different PBAT amounts... 15

Figure S17. TG analysis from the MCs obtained with different DCM amounts.. 16

Table S6. Variation of the viscosity of the emulsions with the increase of IPDI. 16

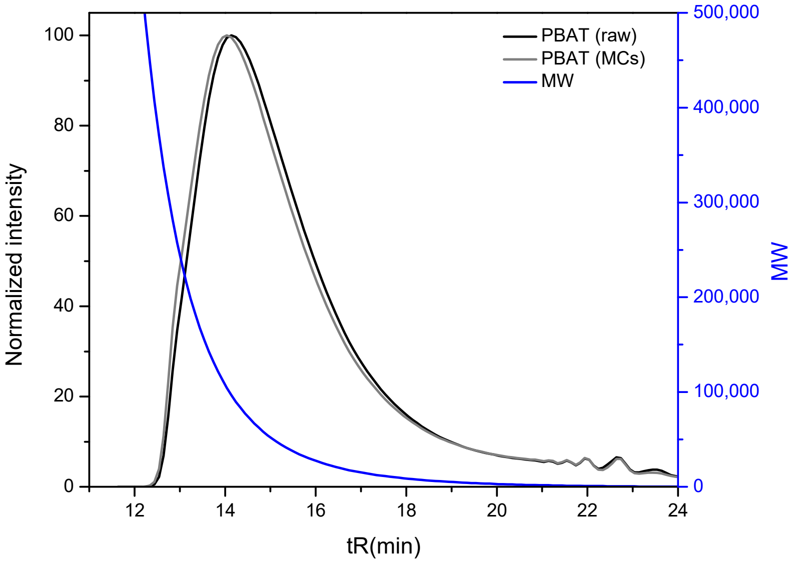

Figure S1. The GPC analysis performed for samples of PBAT, and MCs dissolved in chloroform

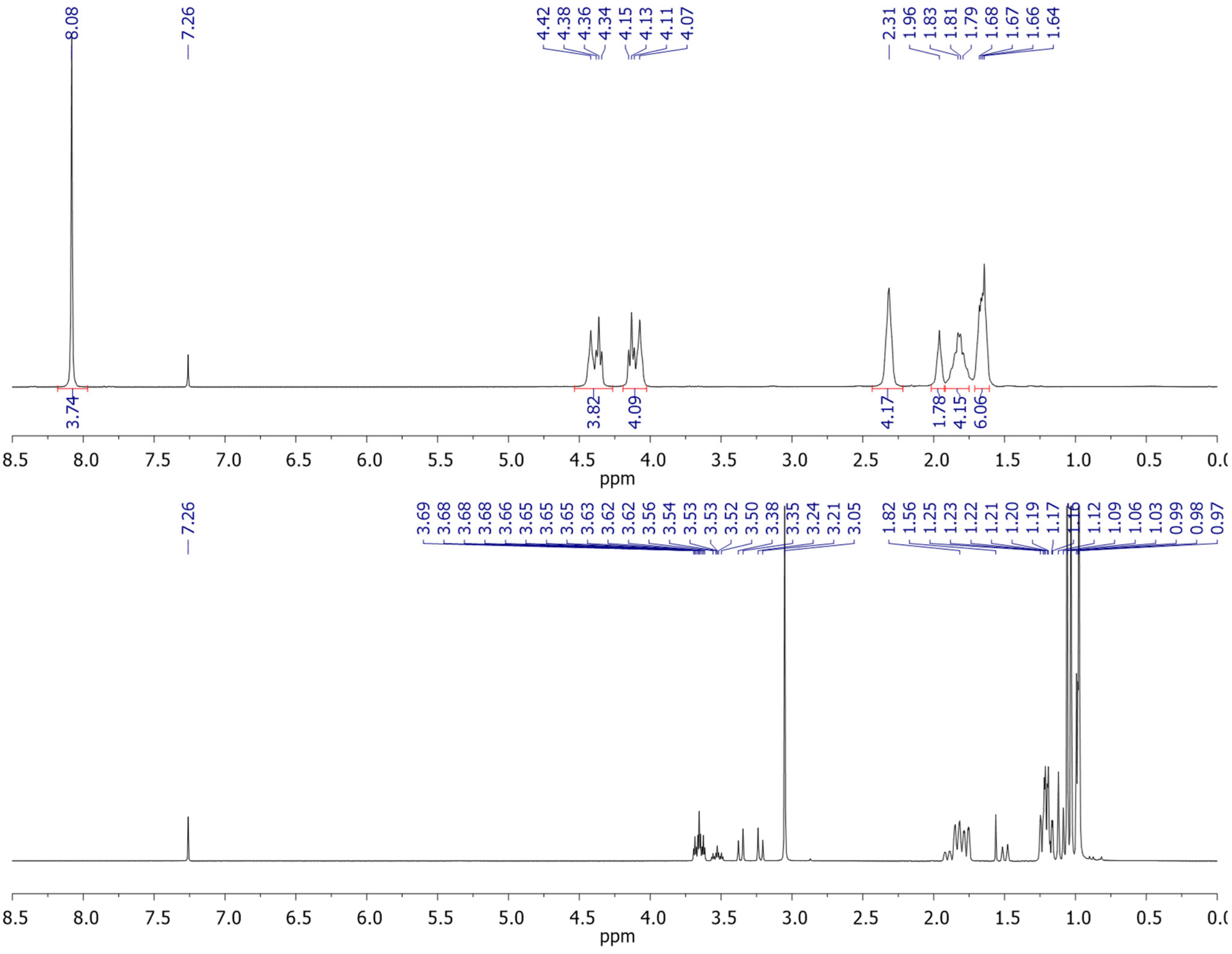

Figure S2. ^{1}H NMR spectra of PBAT (up) and IPDI (down), in $CDCl_3$.

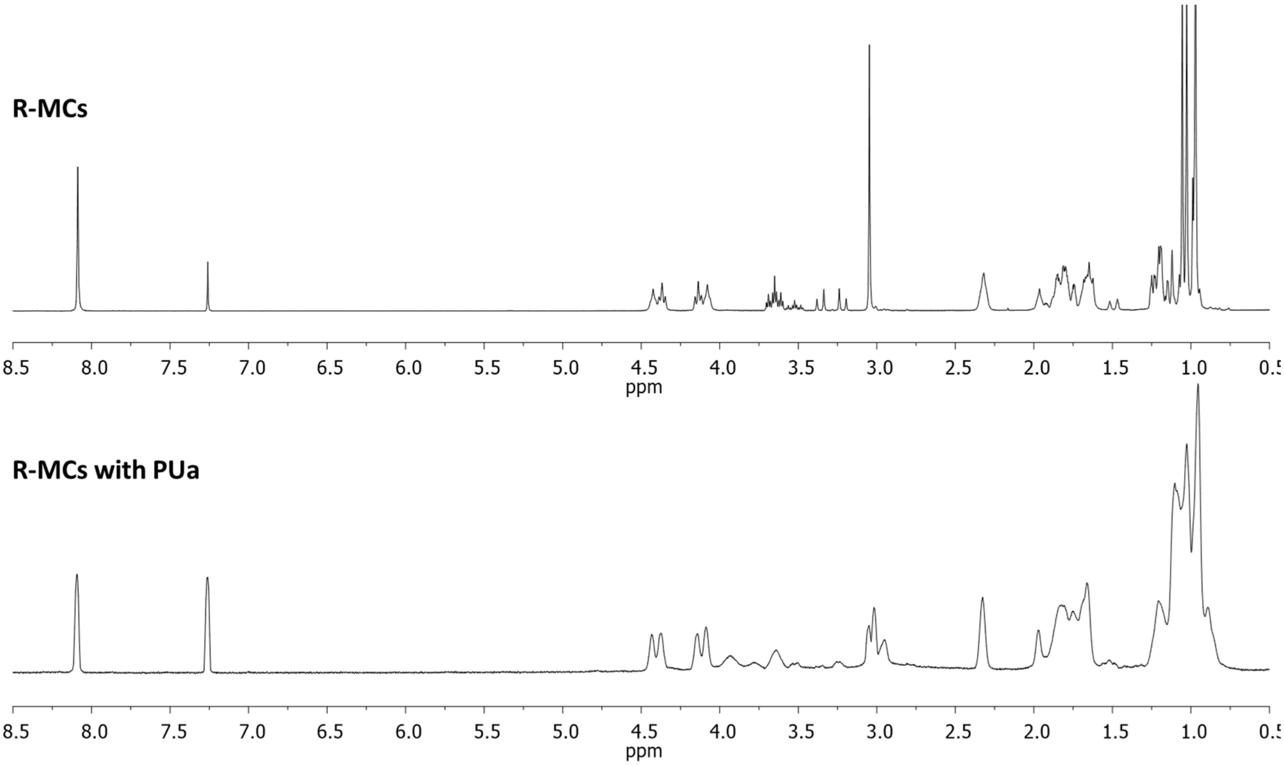

Figure S3. ^{1}H NMR spectra of **R-MCs** and R-MCs with a significant weight percentage of PUa, in $CDCl_3$.

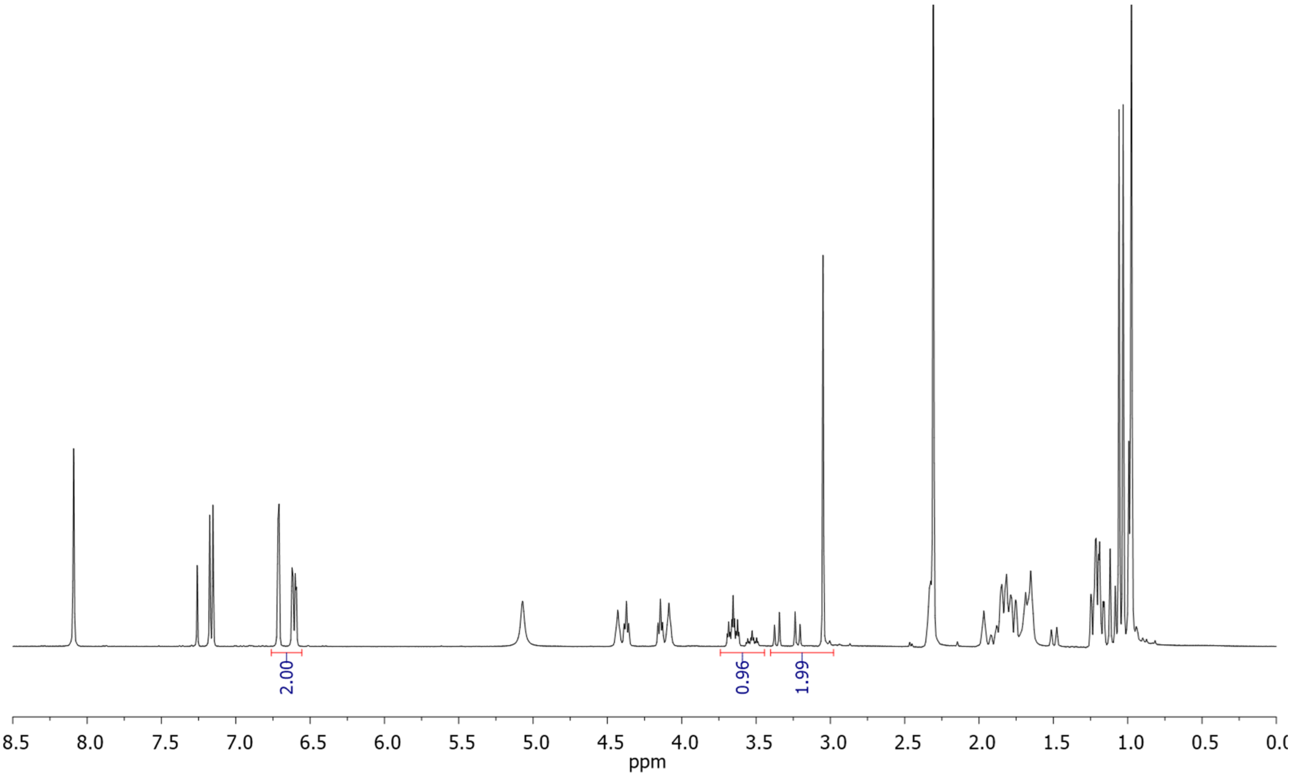

Figure S4. ^{1}H NMR spectra of **R-MCs** (16.5 mg) + 4-chloro-3-methylphenol (7.5 mg), in $CDCl_3$.

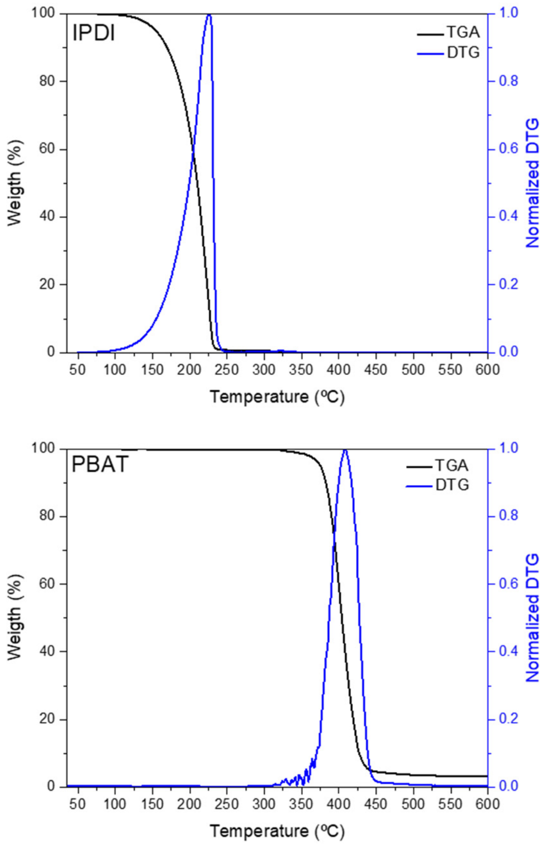

Figure S5. Thermogram and DTG of IPDI and PBAT.

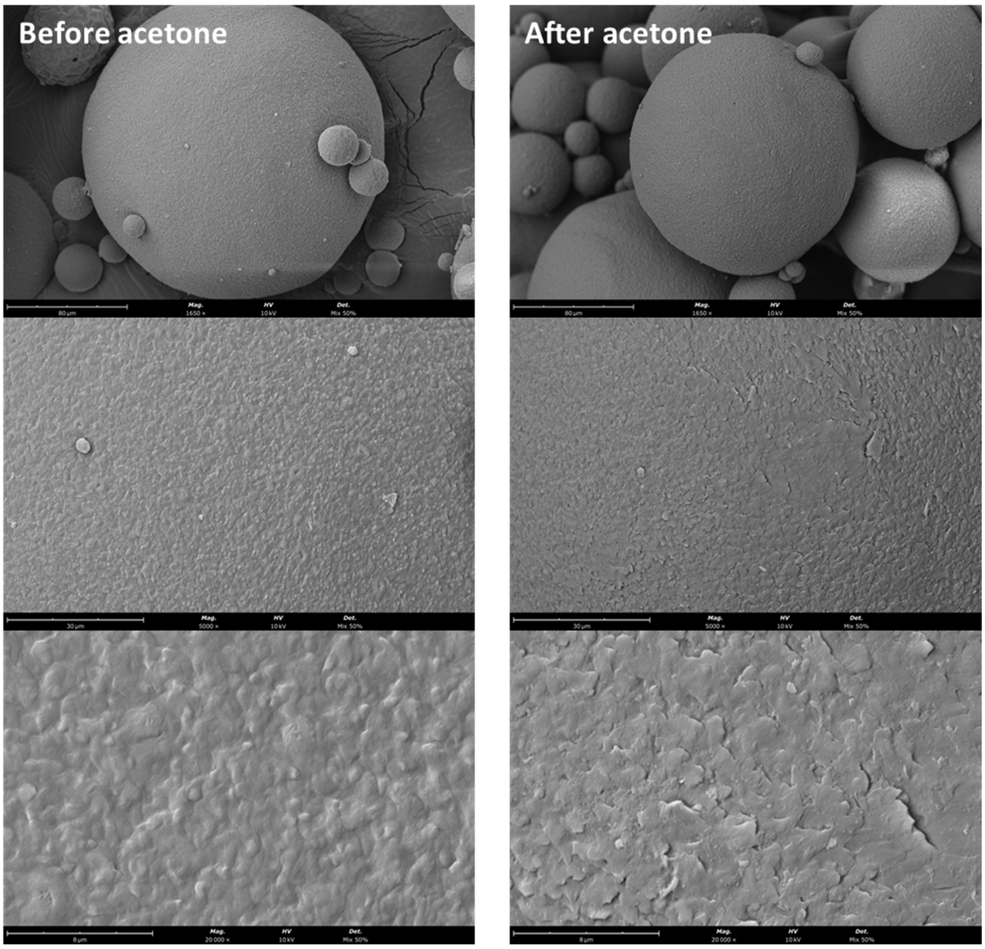

Figure S6. SEM images of the MCs before and after the dispersion in acetone.

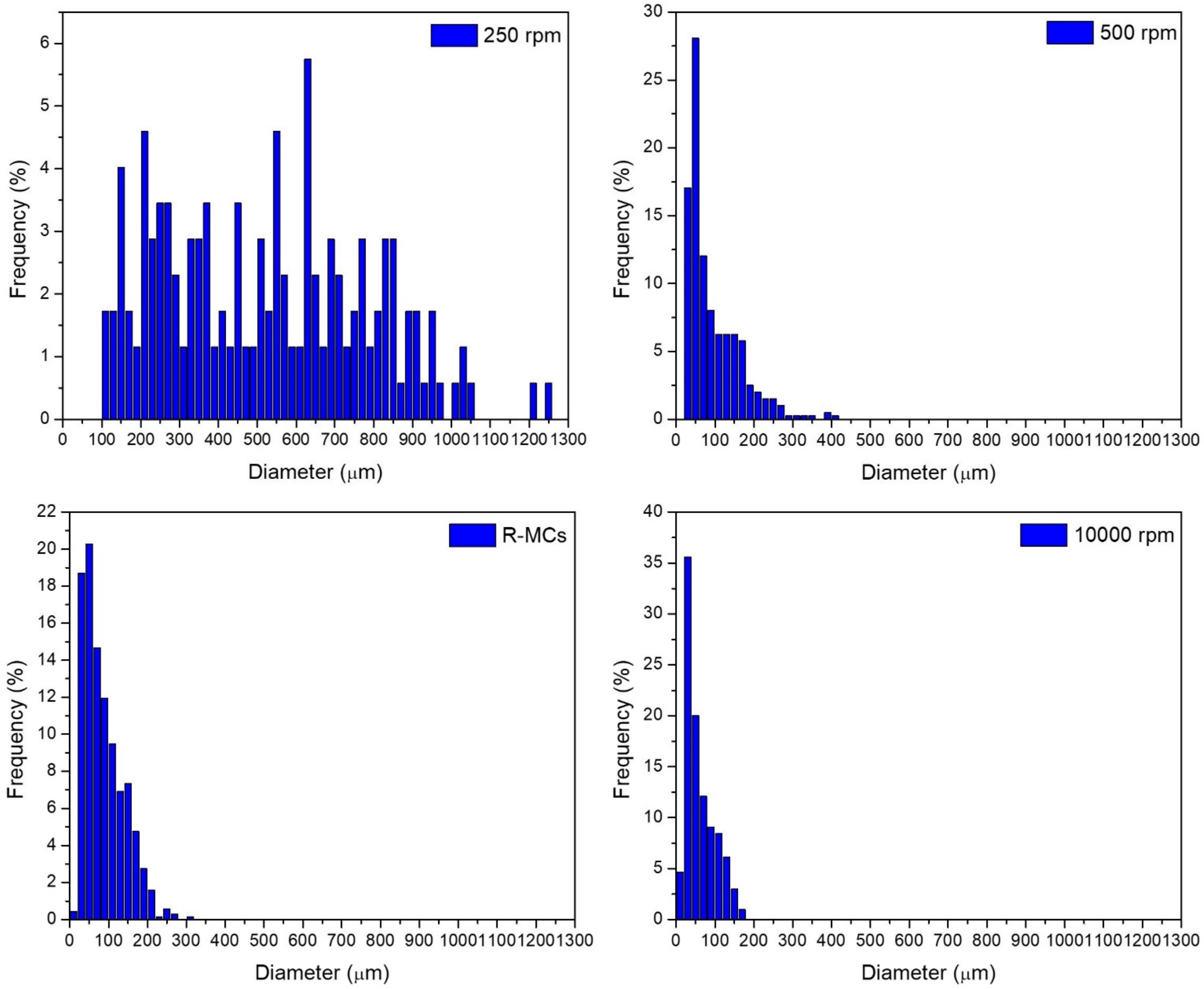

Figure S7. Histogram of the particle size distribution from the MCs obtained under different mixing rates.

Table S1. Set of statistical analysis tables regarding particle size distribution obtained under different mixing rates.

**Descriptive Statistics (stirring rate)**									
**MCs**	**N Analysis**	**Mean**	**Standard deviation**	**SE of Mean**	**Min**	**Q1**	**Median**	**Q3**	**Max**
250 rpm	174	521	521	19.9	106	287	523	718	1249
500 rpm	399	236	236	8.38	50.9	112	166	323	1026
750 rpm	539	107	107	3.03	15.5	48.6	86.2	147	368
1000 rpm	604	61.0	61.0	1.55	8.92	29.9	49.0	85.4	178

**Kruskal–Wallis test (stirring rate)**		
**χ^2 (Chi-Square)**	**DF**	**p**
889	3	<0.001

At least two medians are different ($p < 0.05$).

**Dwass–Steel–Critchlow–Fligner (stirring rate)**		
	**W**	**p**
500 rpm – 250 rpm	-17.9	< 0.001
750 rpm – 250 rpm	-26.0	< 0.001
750 rpm – 500 rpm	-28.2	< 0.001
1000 rpm – 250 rpm	-21.3	< 0.001
1000 rpm – 500 rpm	-32.8	< 0.001
1000 rpm – 750 rpm	-17.5	< 0.001

The difference is significant at the 0.05 level ($p < 0.05$).

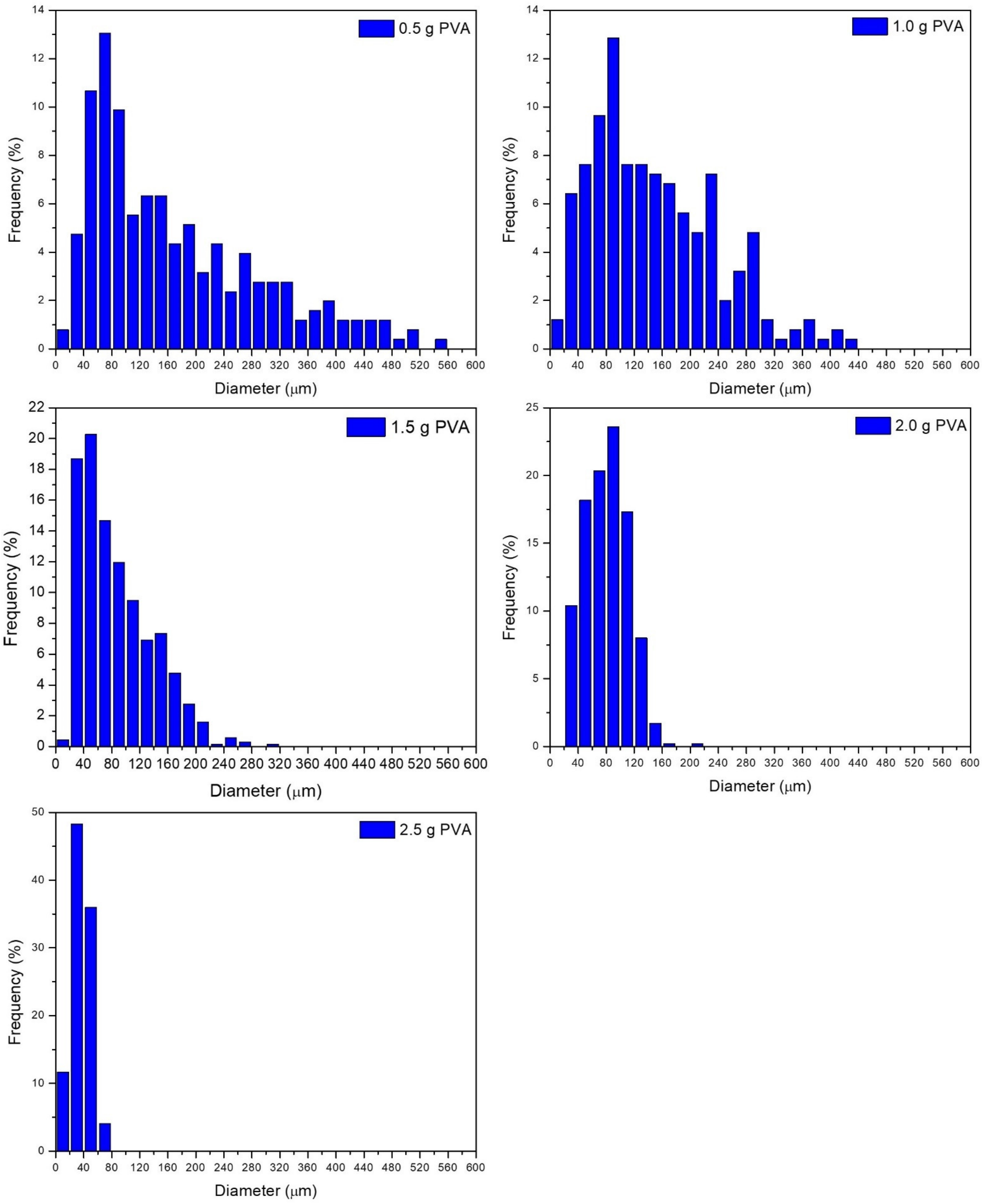

Figure S8. Histogram of the particle size distribution from the MCs obtained with different PVA amounts.

Table S2. Set of statistical analysis tables regarding particle size distribution obtained with different PVA amounts.

**Descriptive Statistics (PVA amount)**									
**MCs**	**N Analysis**	**Mean**	**Standard deviation**	**SE of Mean**	**Min**	**Q1**	**Median**	**Q3**	**Max**
0.5 g	253	171	121	7.63	18.6	73.8	139	242	541
1.0 g	249	149	89.3	5.66	17.1	80.1	133	213	421
1.5 g	539	107	70.3	3.03	15.5	48.6	86.2	147	368
2.0 g	462	80.5	30.3	1.41	21.4	56.3	81.1	102	212
2.5 g	642	36.5	13.5	0.533	8.48	26.2	35.6	46.6	73.9

**Kruskal–Wallis test (PVA amount)**		
**χ^2 (Chi-Square)**	**DF**	**p**
962	4	< 0.001

At least two medians are different ($p < 0.05$).

**Dwass–Steel–Critchlow–Fligner (PVA amount)**		
	**W**	**p**
1.0 g – 0.5 g	-1.52	0.822
1.5 g – 0.5 g	-10.24	<0.001
2.0 g – 0.5 g	-14.19	< 0.001
2.5 g – 0.5 g	-29.02	< 0.001
1.0 g – 1.5 g	-9.24	< 0.001
1.0 g – 2.0g	-14.70	< 0.001
1.0 g – 2.5 g	-28.38	< 0.001
1.5 g – 2.0 g	-5.03	0.003
1.5 g – 2.5 g	-30.58	< 0.001
2.0 g – 2.5 g	-32.68	< 0.001

The difference is significant at the 0.05 level ($p < 0.05$).

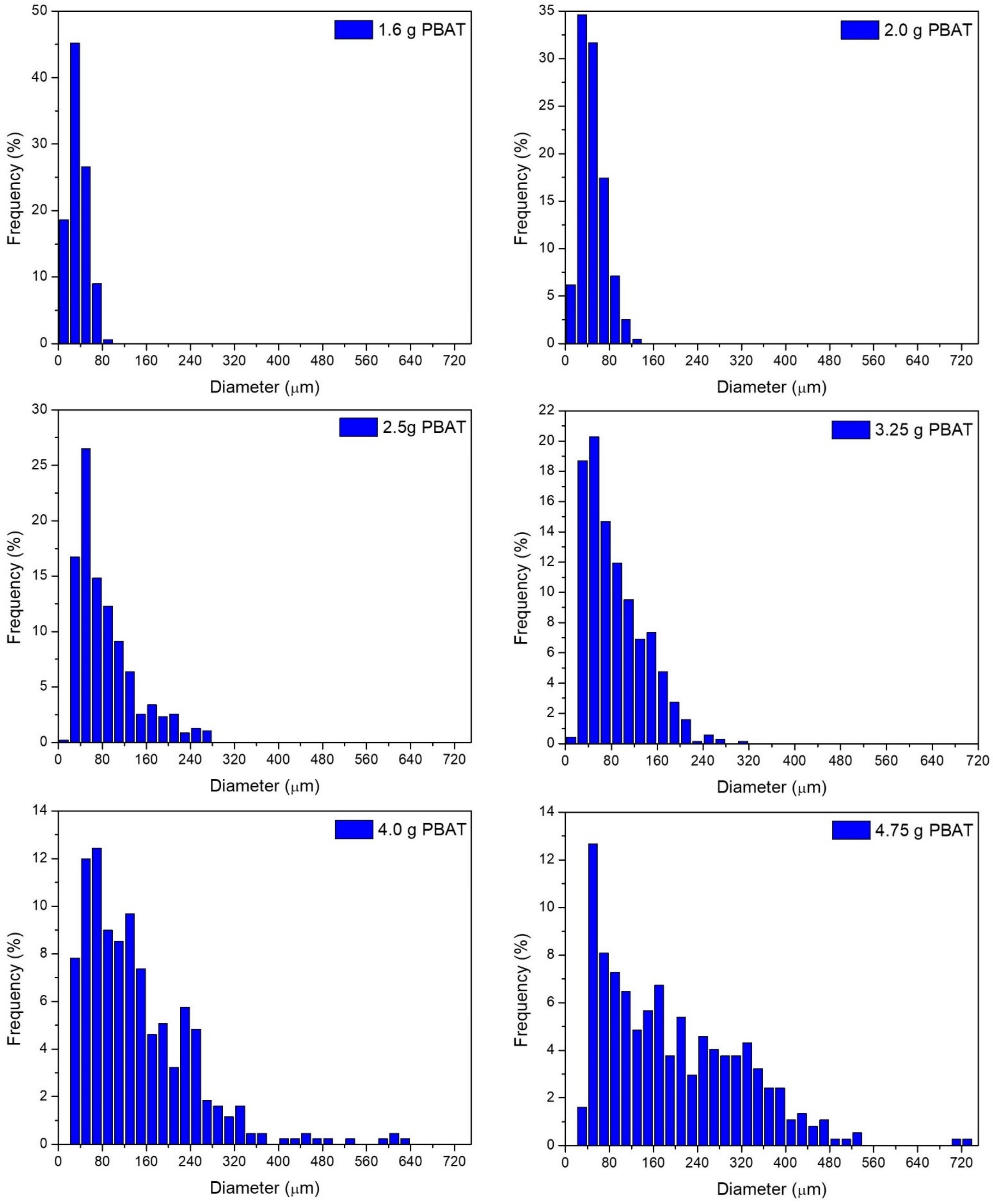

Figure S9. Histogram of the particle size distribution from the MCs obtained with different PBAT amounts.

Table S3. Set of statistical analysis tables regarding particle size distribution obtained with different PBAT amounts.

**Descriptive Statistics (PBAT amount)**									
**MCs**	**N Analysis**	**Mean**	**Standard deviation**	**SE of Mean**	**Min**	**Q1**	**Median**	**Q3**	**Max**
1.6 g	500	35.6	16.1	0.718	8.10	23.1	34.0	46.3	84.2
2.0 g	436	48.6	22.2	1.07	8.25	31.6	44.7	62.2	123
2.25 g	472	85.8	53.9	2.48	17.9	45.8	67.1	109	271
3.25 g	539	107	70.3	3.03	15.5	48.6	86.2	147	368
4.0 g	434	143	99.5	4.77	21.7	69.3	120	191	622
4.75 g	371	195	125	6.51	26.5	86.7	169	284	722

Kruskal–Wallis test **(PBAT amount)**		
**χ² (Chi-Square)**	DF	**p**
1179	5	< .001

At least two medians are different ($p < 0.05$).

**Dwass–Steel–Critchlow–Fligner (PBAT amount)**		
	**W**	**p**
1.6 g – 2.0 g	12.87	<0 .001
1.6 g – 2.5 g	26.99	< 0.001
1.6 g – 3.25 g	29.14	< 0.001
1.6 g – 4.0 g	32.30	< 0.001
1.6 g – 4.75 g	33.17	< 0.001
2.0 g – 2.5 g	17.10	< 0.001
2.0 g – 3.25 g	20.76	< 0.001
2.0 g – 4.0 g	26.68	< 0.001
2.0 g – 4.75 g	28.84	< 0.001
2.5 g – 3.25 g	5.77	< 0.001
2.5 g – 4.0 g	14.55	< 0.001
2.5 g – 4.75 g	20.23	< 0.001
3.25g – 4.0 g	8.76	< 0.001
3.25 g – 4.75	16.25	< 0.001
4.0 g – 4.75 g	8.48	< 0.001

The difference is significant at the 0.05 level ($p < 0.05$).

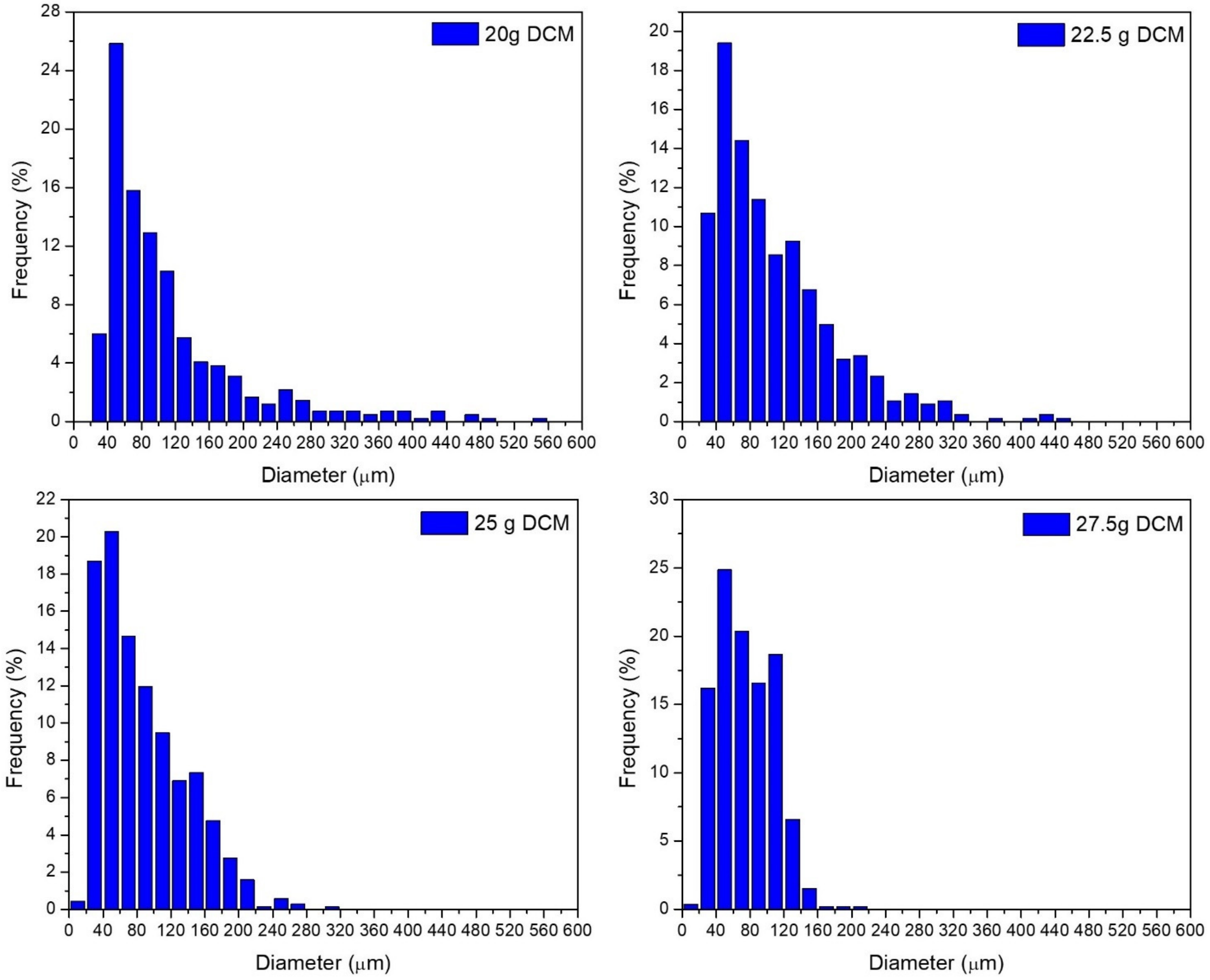

Figure S10. Histogram of the particle size distribution from the MCs obtained with different DCM amounts.

Table S4. Set of statistical analysis tables regarding particle size distribution obtained with different DCM amounts.

**Descriptive Statistics (Amount of DCM)**									
**MCs**	**N Analysis**	**Mean**	**Standard deviation**	**SE of Mean**	**Min**	**Q1**	**Median**	**Q3**	**Max**
20.0 g	418	112	88.1	4.31	25.5	54.9	83.7	132	550
22.5 g	562	109	71.5	3.02	20.6	53.5	91.5	144	441
25.0 g	539	107	70.3	3.03	15.5	48.6	86.2	147	368
27.5 g	561	74.5	32.3	1.36	19.5	46.2	69.6	100	211

**Kruskal–Wallis test (Amount of DCM)**		
**χ^2 (Chi-Square)**	**DF**	**p**
69.5	3	< 0.001

At least two medians are different ($p < 0.05$).

**Dwass–Steel–Critchlow–Fligner (Amount of DCM)**		
	**W**	**p**
20.0 g – 22.5 g	1.135	0.853
20.0 g – 25.0 g	-0.774	0.947
20.0 g – 27.5 g	-8.344	< 0.001
22.5 g – 25.0 g	-1.609	0.666
22.5 g – 27.5 g	-10.964	< 0.001
25.0 g – 27.5 g	-8.605	< 0.001

The difference is significant at the 0.05 level ($p < 0.05$).

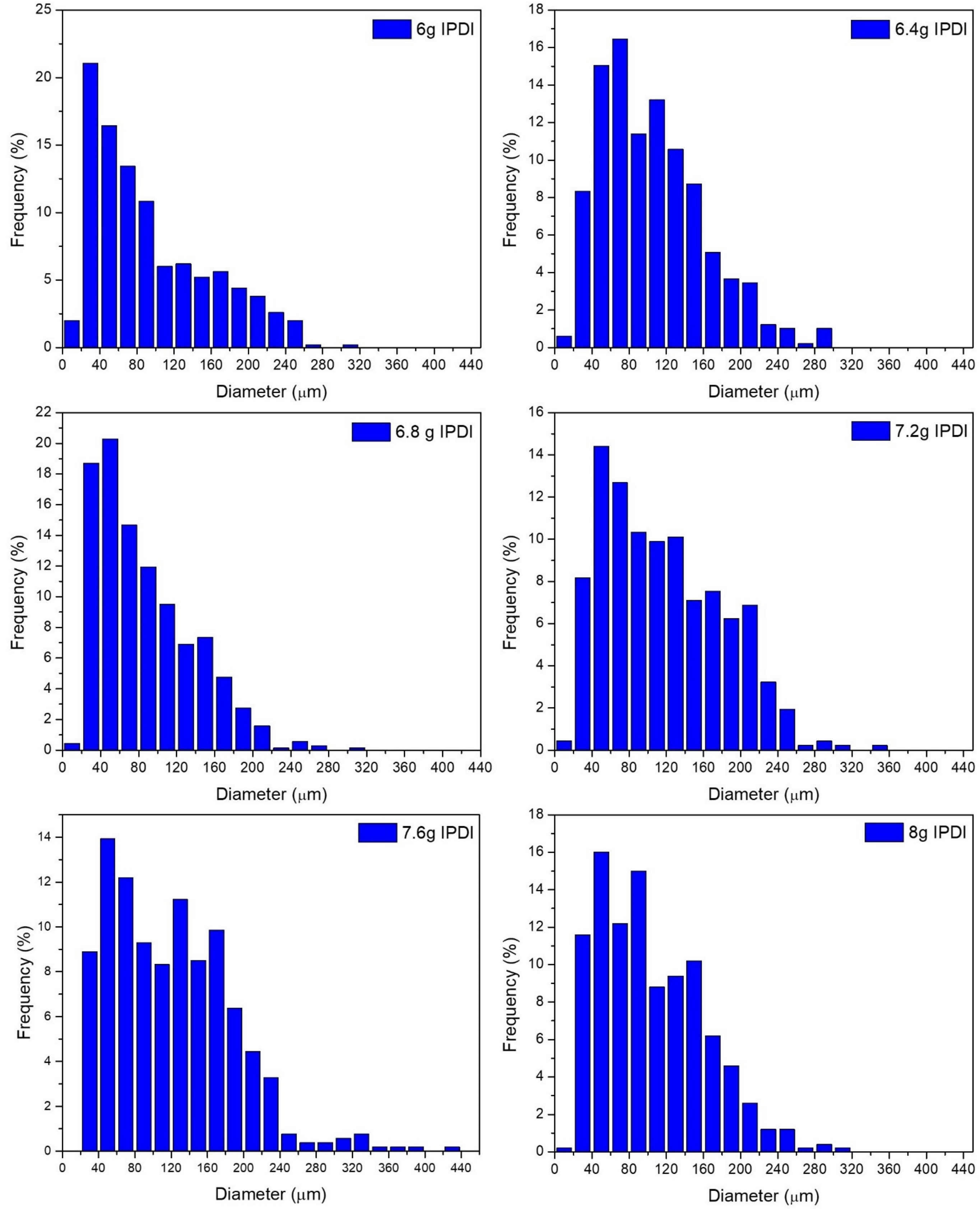

Figure S11. Histogram of the particle size distribution from the MCs obtained with different IPDI amounts.

Table S5. Set of statistical analysis tables regarding particle size distribution obtained with different DCM amounts.

**Descriptive Statistics (Amount of IPDI)**									
**MCs**	**N Analysis**	**Mean**	**Standard deviation**	**SE of Mean**	**Min**	**Q1**	**Median**	**Q3**	**Max**
6.0 g	500	94.3	63.1	2.82	10.2	41.6	75.1	136	304
6.4 g	492	105.1	55.6	2.51	15.6	61.0	97.6	138	299
6.8 g	539	106.6	70.3	3.03	15.5	48.6	86.2	147	368
7.2 g	465	117.3	63.4	2.94	19.3	62.7	107.8	164	354
7.6 g	517	120.2	68.2	3.00	20.3	64.0	115.8	165	435
8.0 g	500	103.8	56.4	2.52	14.3	56.4	94.0	144	315

**Kruskal–Wallis test (Amount of IPDI)**		
**χ^2 (Chi-Square)**	DF	**p**
65.0	5	< 0.001

At least two medians are different ($p < 0.05$).

**Dwass–Steel–Critchlow–Fligner (Amount of IPDI)**		
	**W**	**p**
6.0 g – 6.4 g	6.038	<0 .001
6.0 g – 6.8 g	4.055	0.048
6.0 g – 7.2 g	8.922	< 0.001
6.0 g – 7.6 g	9.385	< 0.001
6.0 g – 8.0 g	5.227	0.003
6.4 g – 6.8 g	-2.042	0.700
6.4 g – 7.2 g	3.713	0.091
6.4 g – 7.6 g	4.503	0.018
6.4 g – 8.0 g	-0.729	0.996
6.8 g – 7.2 g	5.165	0.004
6.8 g – 7.6 g	5.623	< 0.001
6.8 g – 8.0 g	1.161	0.964
7.2 g – 7.6 g	0.404	1.000
7.2. g – 8.0 g	-4.454	0.020
7.6 g – 8.0 g	-5.071	0.005

The difference is significant at the 0.05 level ($p < 0.05$).

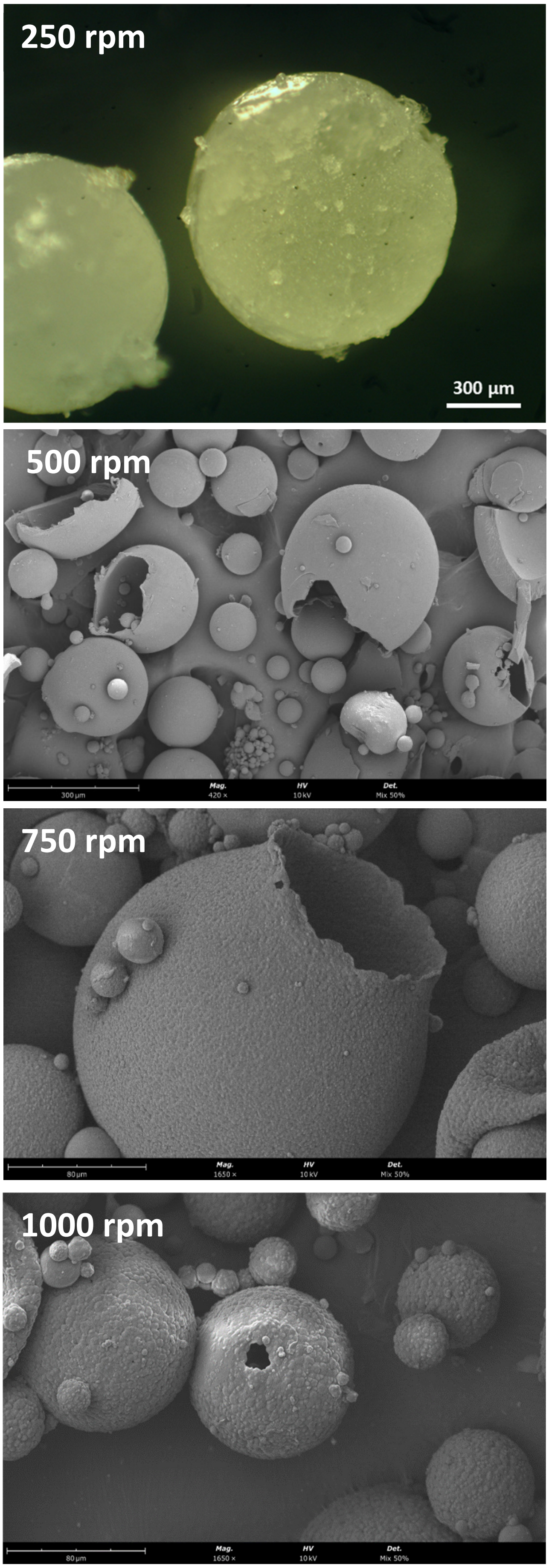

Figure S12. Optical microscopy photograph (250 rpm) and SEM images of the MCs obtained under different mixing rates.

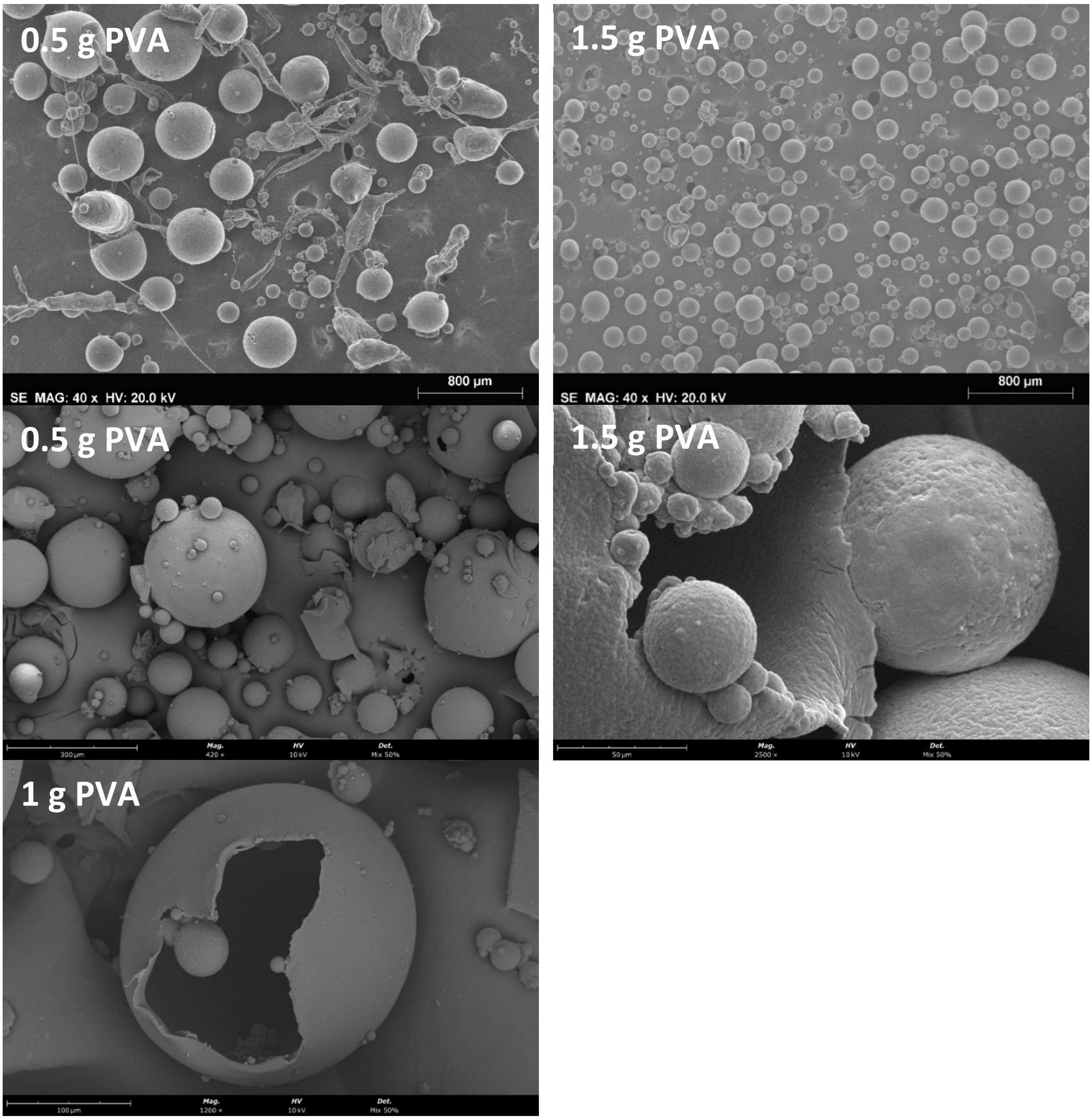

Figure S13. SEM images of the MCs obtained with 0.5, 1 and 1.5 g of PVA

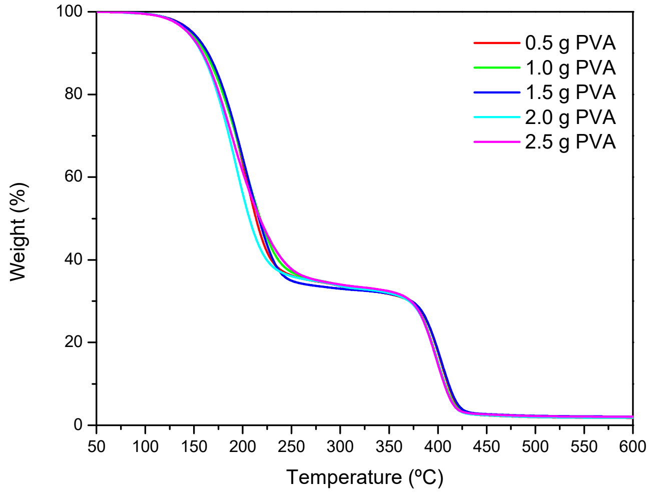

Figure S14. TG analysis from the MCs obtained with different PVA amounts.

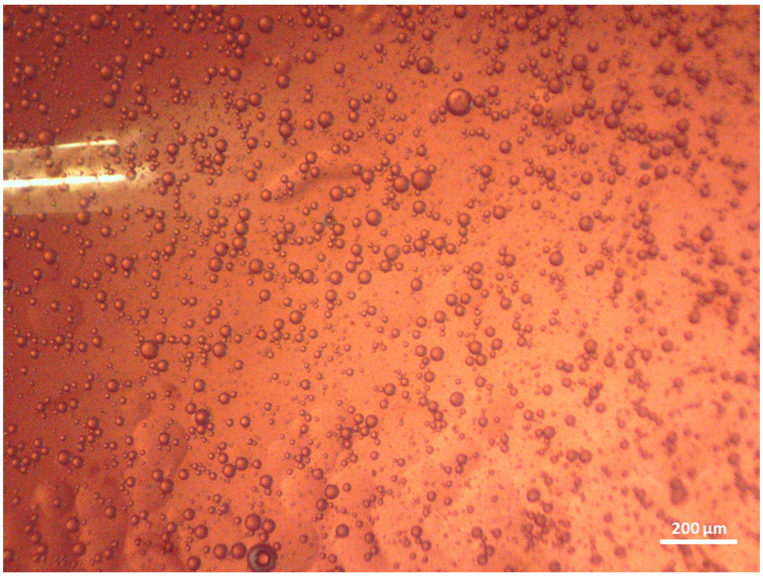

Figure S15. Optical microscopy photograph from the MCs obtained with 2g of PBAT.

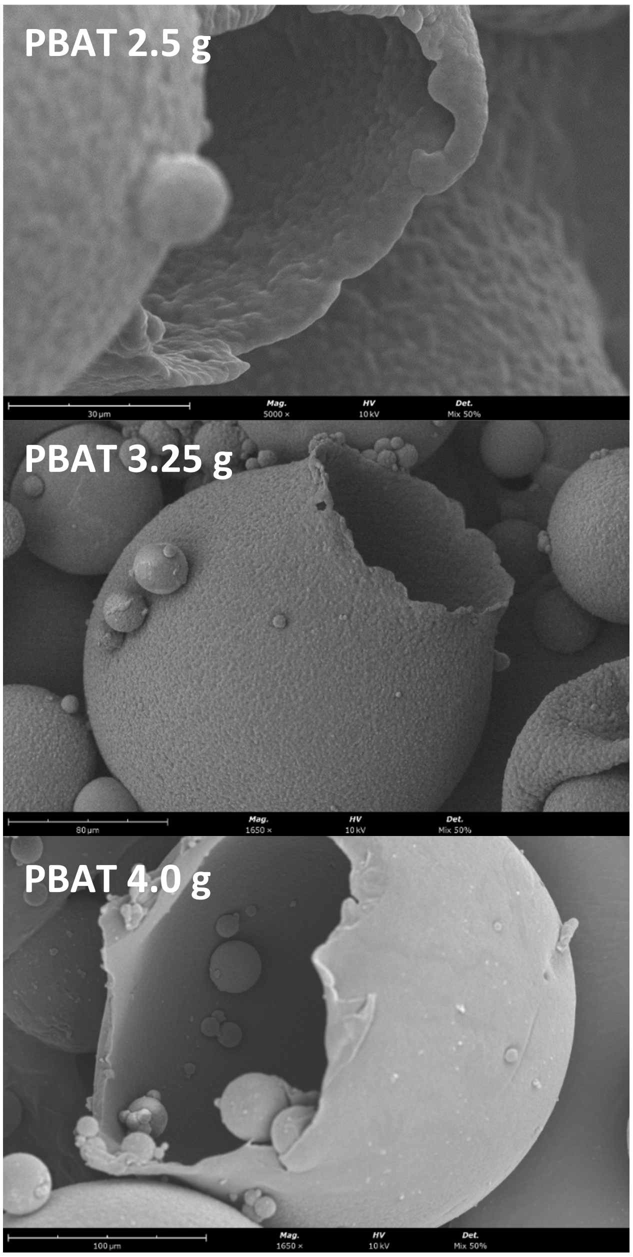

Figure S16. SEM images of MCs obtained with different PBAT amounts.

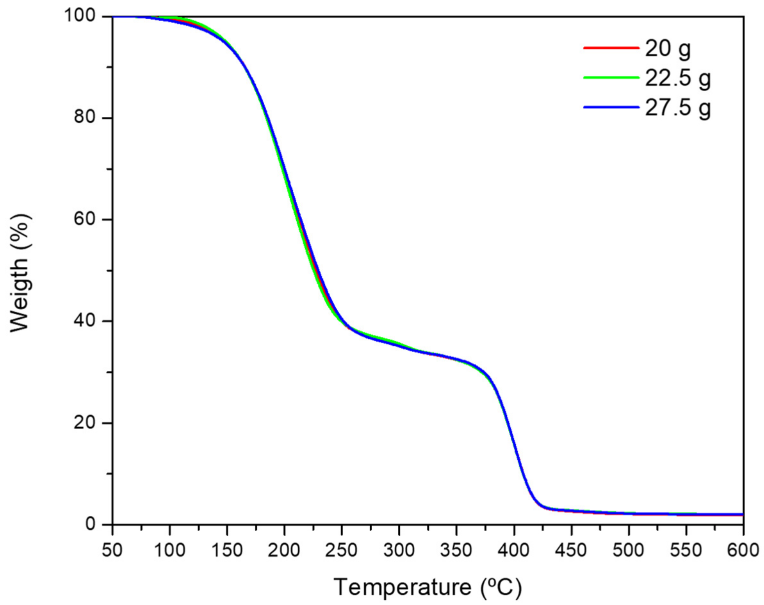

Figure S17. TG analysis from the MCs obtained with different DCM amounts.

Table S6. Variation of the viscosity of the emulsions with the increase of IPDI.

IPDI(g)	Emulsion viscosity (cP)	Mean size (µm)
6.00	75.83 ± 1.72	94 ± 63
6.40	68.53 ± 0.28	105 ±56
6.80	61.35 ± 0.71	107 ± 70
7.20	54.04 ± 1.21	117 ± 63
7.60	49.12 ± 1.25	120 ± 68
8.00	40.82 ± 0.29	104 ± 56
